# Electron‐Induced Molecular Programming Drives Interfacial Chemistry for Ah‐Level Zinc Batteries

**DOI:** 10.1002/adma.72891

**Published:** 2026-03-24

**Authors:** Feifei Wang, Yuhang Zhuang, Jiwei Shi, Haojie Zhang, Peng Zhang, Songshan Bi, Hyejung Yang, Wenqiang Yang, Stuart S. P. Parkin, Chunpeng Yang, Quan‐Hong Yang, Ali Shaygan Nia, Xinliang Feng

**Affiliations:** ^1^ Max Planck Institute For Microstructure Physics Halle (Saale) Germany; ^2^ Center for Advancing Electronics Dresden (cfaed) and Faculty of Chemistry and Food Chemistry Technische Universität Dresden Dresden Germany; ^3^ Nanoyang Group Tianjin Key Laboratory of Advanced Carbon and Electrochemical Energy Storage School of Chemical Engineering and Technology and National Industry‐Education Integration Platform of Energy Storage Tianjin University Tianjin China

## Abstract

Solid–electrolyte interphases (SEIs) are essential for stabilizing metal anodes in aqueous zinc (Zn) batteries (AZBs), yet their formation remains intrinsically uncontrolled, leaving the interphase vulnerable to dissolution and water‐driven parasitic reactions. Herein, we report an electron‐induced molecular programming strategy that uses only 1 mM of 4‐bromobenzenediazonium tetrafluoroborate (BDTF) to in situ construct a Zn^2+^‐favored molecular lock on the ZnF_2_‐rich SEI surface. Electrochemically generated *p*‐bromoaniline becomes molecularly woven into the inorganic layer, forming an ultrathin molecular‐lock shell (∼1 nm) atop a graded hybrid SEI. Through N–Zn coordination coupled with Br‐induced interfacial polarization, the molecular lock reorganizes the local electrostatic environment, stabilizes ZnF_2_, limits water access, and promotes desolvation‐facilitated Zn^2+^ transport. As a result, the programmed SEI enables highly reversible Zn plating/stripping with a 99.8% average Coulombic efficiency, and stable cycling under 80% depth of discharge at 10 mA cm^−^^2^. Moreover, it displays broad cathode compatibility, extending cycling stability in vanadium‐, manganese‐, and iodine‐based full cells. In Ah‐level pouch cells with ultrahigh vanadium‐based cathode loading (21 mg cm^−2^), the system delivers 1.2 Ah with 81% retention after 100 cycles, surpassing state‐of‐the‐art aqueous Zn batteries that typically fail at high mass loading.

## Graphical abstract

We establish an electron‐driven molecular programming strategy that converts spontaneous solid electrolyte interphase formation into a bonding‐defined interfacial architecture. Coordinative integration of *p*‐bromoaniline with ZnF_2_ forms a dipole‐regulated molecular lock that reconfigures interfacial electrostatics, suppresses parasitic water reactions, and directs Zn^2+^ transport, enabling durable and scalable Ah‐level aqueous Zn batteries.

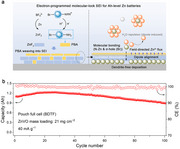

## 1 Introduction

**SCHEME 1:**
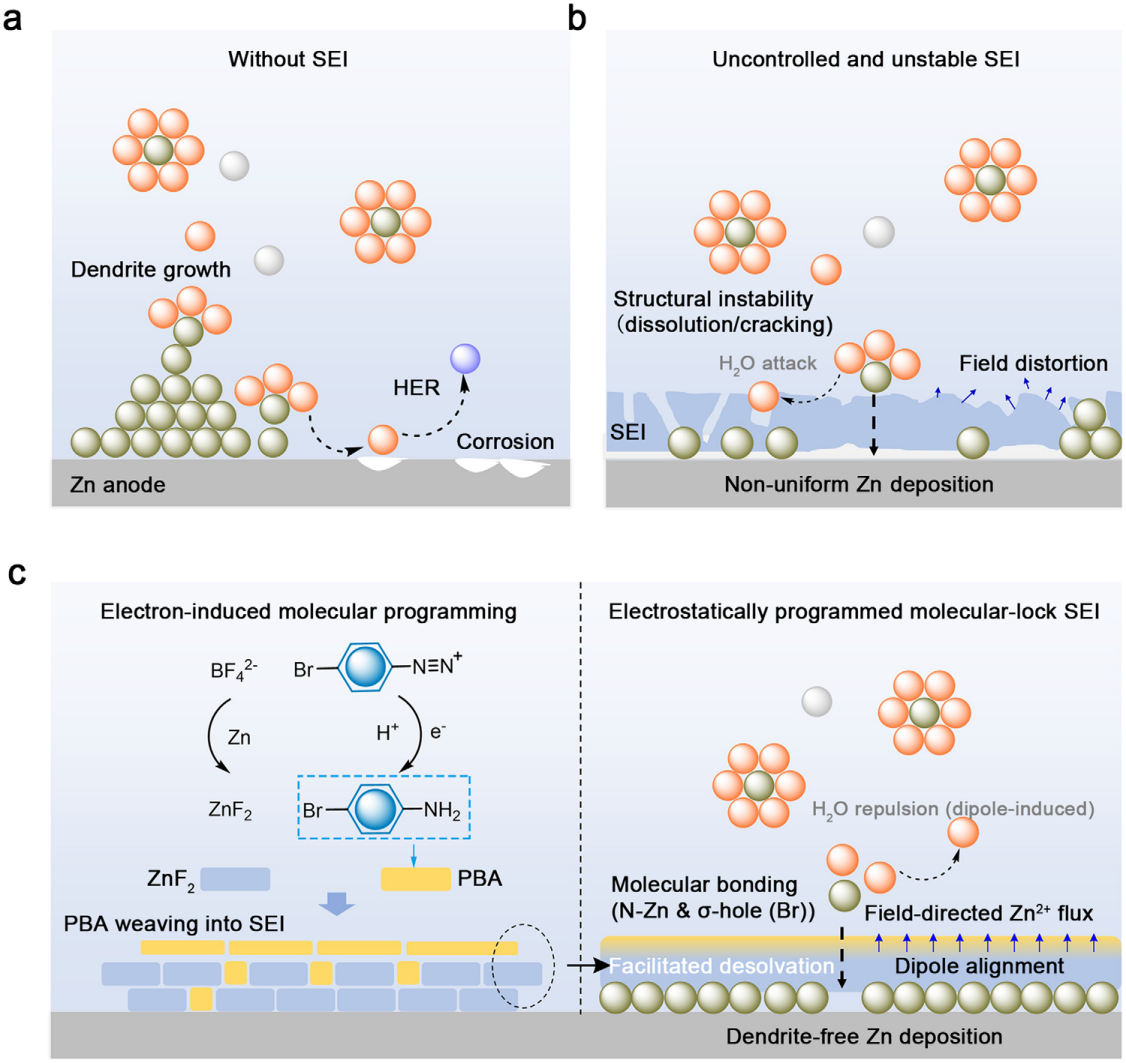
Conceptual illustration of electron‐induced molecular programming for constructing a graded molecular‐lock SEI. (a) Without an effective SEI, the Zn anode suffers from uncontrolled dendrite growth, HER, and corrosion arising from interfacial instability. (b) Conventional hybrid SEIs formed by stochastic interfacial reactions exhibit structural instability (dissolution and cracking), and nonuniform Zn^2+^ transport due to uncontrolled electric‐field distortion. (c) Electron‐induced molecular programming of BDTF generates PBA and ZnF_2_ in situ, which cooperatively assemble into a graded molecular‐lock SEI. The ultrathin organic lock chemically stabilizes ZnF_2_, repels interfacial H_2_O, and electrostatically aligns interfacial dipoles to regulate the local electric field. This programmed interphase accelerates Zn^2+^ desolvation and directs uniform Zn^2+^ flux, ultimately enabling dendrite‐free Zn deposition (orange: H_2_O; green: Zn^2+^; purple: H_2_; grey: SO_4_^2−^).

Aqueous zinc (Zn) batteries (AZBs) are considered among the most promising energy storage systems for grid‐scale energy storage and wearable electronics owing to their high theoretical capacity, intrinsic safety, environmental friendliness, and low cost [1, 2, 3]. Despite these advantages, their commercialization is critically hindered by uncontrolled dendritic Zn growth and parasitic side reactions (e.g., hydrogen evolution and corrosion) at the Zn‐electrolyte interface (Scheme 1a) [4, 5]. The formation of dendrites is mainly attributed to uneven Zn^2+^ flux, local electric field amplification, and surface energy anisotropy, which results in tip growth instability and interfacial breakdown. These detrimental processes originate from the intrinsically uncontrolled formation and instability of the solid–electrolyte interphases (SEIs), which degrade the reversibility of Zn plating/stripping and raise severe safety concerns, thus limiting the practical application of AZBs [6, 7].

Constructing artificial SEIs has therefore been widely explored to stabilize Zn surfaces [8, 9, 10, 11, 12, 13, 14, 15, 16, 17]. In particular, hybrid SEIs combining inorganic components (e.g., ZnF_2_, Zn_3_(PO_4_)_2_, ZnS) [18, 19, 20, 21] and organic modifiers (e.g., polymers, small molecules) [22, 23, 24] aim to couple mechanical robustness with chemical protection [25, 26]. However, many reported hybrid SEIs primarily originate from additive decomposition or solvation restructuring, where the structure emerges as a consequence of interfacial reactions rather than structurally defined molecular assembly (Table S1) [27, 28]. Although such strategies can effectively suppress parasitic reactions, the resulting interphases are primarily composition‐driven, with limited control over interfacial bonding configuration and electrostatic environment. This formation paradigm may lead to structural heterogeneity, reduced interfacial stability in aqueous media, and dependence on relatively high additive concentrations (>10 mm), which can increase resistance and compromise Zn^2+^ transport selectivity (Scheme 1b) [8, 23, 29, 30]. Therefore, beyond compositional passivation, a bonding‐defined and electrostatically programmable interphase remains highly desirable for achieving stable and directional Zn^2+^ regulation.

To overcome these limitations, herein we propose an electron‐induced molecular programming strategy that in situ constructs a Zn^2+^‐favored molecular‐lock interphase. This molecular lock forms an ultrathin (∼1 nm) organic outer layer stabilized by PBA–ZnF_2_ coordination, thereby establishing a dipole‐regulated structure with compositional gradients and interfacial field‐control capability (Scheme 1c). By introducing an ultra‐lean concentration (1 mm) of 4‐bromobenzenediazonium tetrafluoroborate (BDTF) additives, electron‐triggered reduction generates high‐dipole PBA, which becomes molecularly interwoven into a graded SEI featuring an outer layer atop a ZnF_2_‐rich inner domain. The resulting molecular‐lock SEI, established through N–Zn coordination and reinforced by Br‐induced σ‐hole polarization, stabilizes ZnF_2_ against dissolution, embeds persistent dipoles to reshape the interfacial electrostatic environment, guides Zn^2+^ migration, and repels interfacial water to suppress the hydrogen evolution reaction (HER). As a result, Zn symmetric cells with the molecular‐lock SEI achieve stable cycling under 80% depth of discharge (DOD) at 10 mA cm^−2^. Moreover, the molecular‐lock SEI exhibits broad compatibility across diverse cathode chemistries, enabling significantly extended full‐cell lifetimes, including 2000‐cycle retention of 99.3% in Mn‐based cells and 50,000‐cycle durability with 84% retention in Zn–I_2_ systems. In pouch‐cell configurations with ultrahigh cathode loading (21 mg cm^−2^), the molecular‐lock SEI supports Ah‐level cycling (1.2 Ah over 100 cycles) with only 1 mm additive, demonstrating clear advantages over conventional composition‐driven SEI strategies. Collectively, these results highlight the distinctive advantage of our molecular programming approach for interfacial regulation in advanced AZBs.

## 2 Results and Discussion

Upon introducing BDTF into the ZnSO_4_ aqueous electrolyte, the interfacial electron‐reduction reaction triggers the programmed formation of a molecular‐lock SEI, in which PBA species and newly formed ZnF_2_ are assembled cooperatively on the Zn surface. The formation proceeds through two coordinated reaction pathways: the 4‐bromobenzenediazonium (BBDZ) cation is electrochemically reduced to PBA that anchors onto freshly deposited Zn, while the BF4^−^ counterion decomposes under reductive conditions, generating F^−^ that reacts with Zn to produce ZnF_2_. Collectively, these reactions establish a vertically graded interphase, with PBA enriched at the outer surface and ZnF_2_ forming a compact sub‐surface layer (Figure S4). This vertically organized structure forms a functionally graded molecular‐lock interface: PBA regulates the surface charge environment and local solvation, while ZnF_2_ provides a stable inner layer that guides Zn^2+^ transport.

**FIGURE 1:**
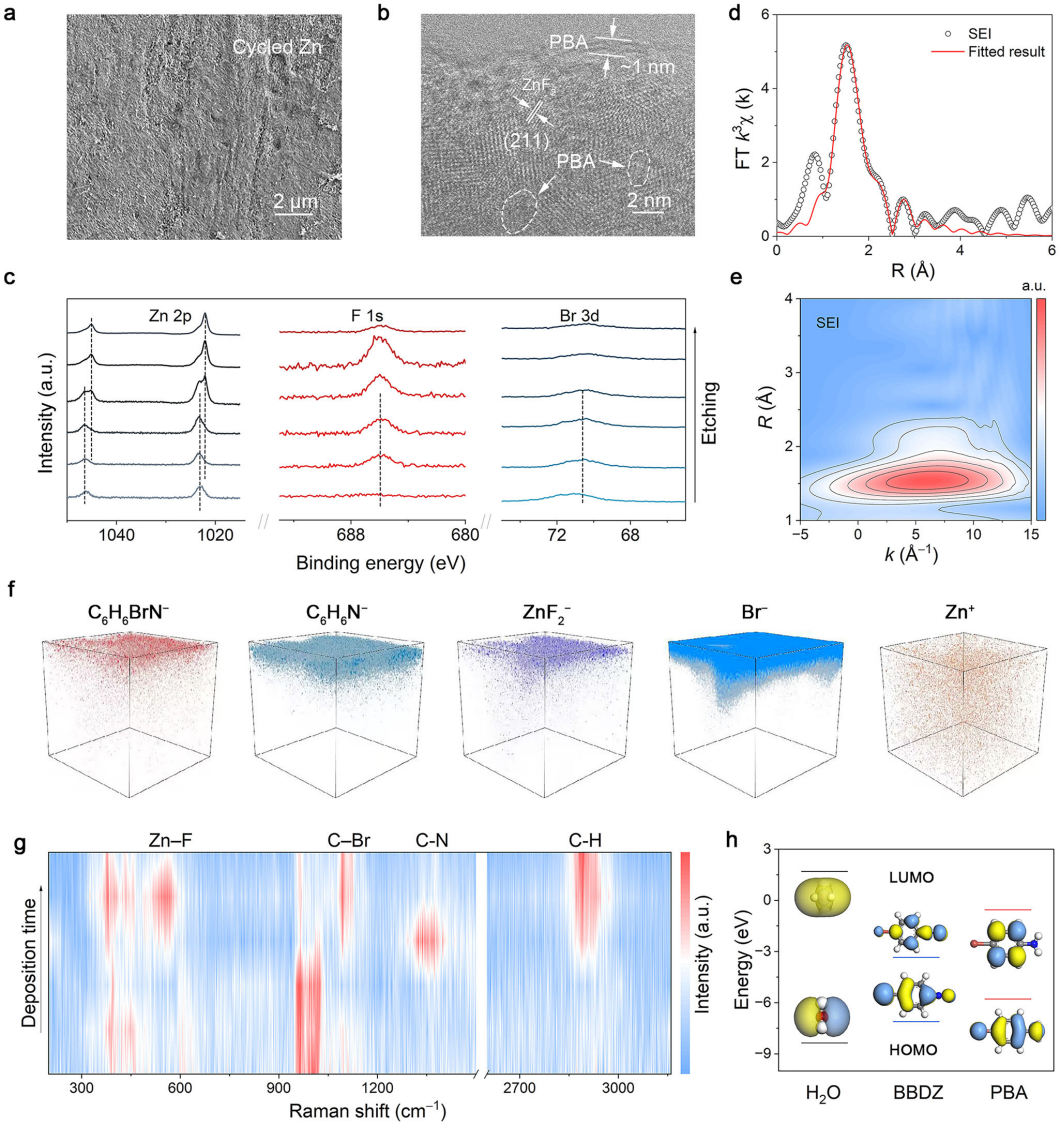
Characterization of the in situ formed hybrid SEI layer on Zn anode in BDTF‐containing electrolyte. (a) SEM image of the cycled Zn surface. (b) HRTEM of the SEI layers on the Zn surface. (c) Depth‐resolved XPS profiles. (d) Fourier‐transformed EXAFS spectra with fitted results. (e) Wavelet transform contour of the Zn K‐edge EXAFS. (f) ToF–SIMS 3D elemental mapping of representative organic and inorganic SEI fragments. (g) in situ Raman spectra to reveal the formation process of the SEI layer. (h) HOMO–LUMO energy levels and orbital distributions of BBDZ, H_2_O, and PBA.

To elucidate the structural characteristics of this molecular‐lock SEI, we conducted comprehensive microscopic and spectroscopic analyses. Scanning electron microscopy (SEM) images of cycled Zn electrodes reveal that the BDTF additive induces a markedly flatter and more compact Zn surface, indicative of a stabilized interphase morphology (Figure 1a). Energy‐dispersive X‐ray spectroscopy (EDS) mapping further confirms the presence of N, Br, and F elements on the Zn surface (Figure S5a), consistent with the reduction of the diazonium precursor and incorporation of its decomposition fragments into the SEI. Transmission electron microscopy (TEM) images further visualize the SEI layer with a thickness of ∼20 nm (Figure S5b). High‐resolution TEM images reveal embedded crystalline domains indexed to the (211) planes of ZnF_2_ [31, 32], embedded in an amorphous interfacial region. An ultrathin organic‐enriched layer with a characteristic thickness on the order of ∼1 nm is observed at the outermost interface, consistent with the presence of electrochemically generated PBA species (Figure 1b).

XPS depth profiling further confirms this vertically graded structure, showing enrichment of Br elements in the outer layers, while Zn signals increase progressively with etching depth (Figure 1c and Figure S5c). In addition, high‐resolution N 1s spectra during depth profiling show the absence of the –N≡N^+^ signal and the emergence of the –NH_2_ signal (Figure S5d) [33], providing direct evidence for the electrochemical reduction of BBDZ to PBA. Complementary Fourier‐transform infrared (FTIR) spectroscopy also confirms the incorporation of PBA, showing characteristic N–H, C–N, and C–Br vibrations, together with the disappearance of the –N≡N^+^ stretching band (Figure S5e).

To resolve the local Zn coordination environment within this graded interphase, we performed X‐ray absorption fine structure (XAFS) analysis. The XANES edge position of the SEI‐protected Zn lies between metallic Zn and ZnCO_3_, consistent with a partially oxidized Zn^2+^ species characteristic of Zn–F coordination (Figure S5f). Fourier‐transformed EXAFS and wavelet transform analyses (Figure 1d,e and Table S2) show a dominant peak at ∼2.0 Å and a strong k‐dependent intensity around 9 Å^−1^, features characteristic of Zn–F coordination rather than Zn–Zn or Zn–O scattering. The absence of long‐range coordination features indicates that ZnF_2_ adopts a locally disordered, nanoscale‐confined structure within the SEI rather than forming bulk crystalline ZnF_2_.

To further resolve the spatial distribution of SEI components, three‐dimensional time‐of‐flight secondary ion mass spectrometry (ToF–SIMS) mapping was performed (Figure 1f). Organic fragments (C_6_H_6_BrN^−^, C_6_H_6_N^−^) are predominantly located in the outer interfacial region, whereas inorganic species such as ZnF_2_^−^ and Zn^+^ are enriched beneath the surface, establishing a clear vertically graded chemical distribution. This spatially coupled distribution supports the concurrent formation of PBA species and ZnF_2_ domains at the Zn interface during electrochemical reduction, consistent with the molecular‐lock SEI architecture.

To track the dynamic formation of the SEI during cycling, we performed in situ Raman spectroscopy (Figure 1g). As Zn deposition proceeds, characteristic peaks of Zn–F, C–Br, C–N, and C–H gradually intensify, indicating the concurrent growth of inorganic and organic components. The disappearance of the –N≡N^+^ vibration and emergence of N–H modes further confirm the stepwise reduction of the diazonium cation to –NH_2_‐terminated PBA that associates with the ZnF_2_ surface (Equation 1). Meanwhile, the decomposition of the BF_4_^−^ counterion releases F^−^ ions, which react with Zn electrode to form ZnF_2_ [34], providing the inorganic component of the growing SEI.

(1) $$p-\mathrm{BrPh}{N_{2}}^{+}+2e^{-}+2H^{+}\to p-\mathrm{BrPhN}H_{2}+N_{2}$$

In situ UV–vis spectroscopy reveals a progressive decrease in the characteristic absorption of BDTF with cycling, indicating its continuous electrochemical consumption (Figure S6a,b). Consistently, gas chromatography–thermal conductivity detector (GC–TCD) analysis of the headspace gases reveals an increased N_2_ signal after cycling, verifying the reduction of diazonium cations to aryl amine species (Figure S6c, d). In addition, mass spectrometry of the SEI extracts identifies PBA as the dominant organic product, confirming the electrochemical reduction of BDTF to amine‐containing species (Figure S7).

To rationalize the preferential reduction and early‐stage assembly processes observed experimentally, we performed density functional theory (DFT) calculations. The lowest unoccupied molecular orbital (LUMO) energy of BBDZ is substantially lower than that of H_2_O and PBA (Figure 1h), indicating that BBDZ is thermodynamically more susceptible to electron uptake during Zn deposition and therefore selectively participates in the initial SEI‐forming reduction. The terminal diazonium nitrogen shows the highest Fukui function value (*f ^−^* = 0.224) (Figure S8), identifying it as the most reactive site for preferential electron acceptance—consistent with the experimentally observed disappearance of the –N≡N^+^ signature. This preferential electron capture triggers diazonium cleavage and produces PBA species that anchor onto freshly deposited Zn, forming the nascent organic layer of the SEI. Consistent with this mechanism, adsorption‐energy calculations show that PBA binds favorably to metallic Zn (Figure S9), supporting its role as the primary organic component deposited during the earliest stage of SEI formation.

**FIGURE 2:**
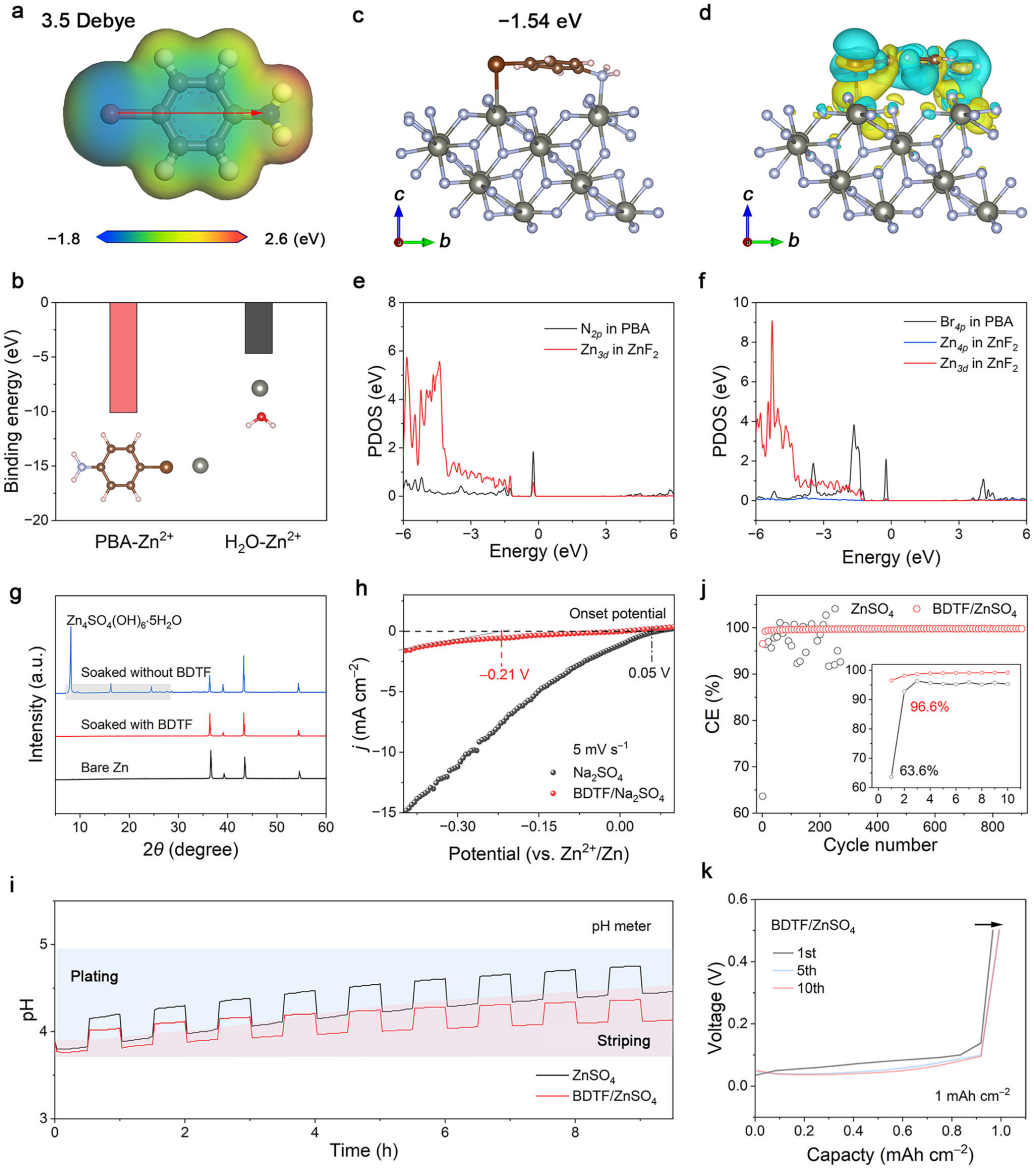
Theoretical and experimental evidence revealing the interfacial stabilization mechanism of the molecular‐lock SEI. (a) ESP map of the PBA molecule with a calculated dipole moment of 3.5 D. (b) Calculated binding energies of PBA–Zn^2+^ and H_2_O–Zn^2+^ complexes. (c) Optimized adsorption configuration of PBA on the ZnF_2_ (211) surface (side view). (d) Charge density difference at the PBA/ZnF_2_ interface. PDOS of (e) N*_2p_* and (f) Br*_4p_* orbitals with ZnF_2_ surface states. (g) XRD patterns of Zn electrodes after static immersion in different electrolytes. (h) LSV curves. (i) pH variation during Zn plating and stripping. (j) CE of Zn plating/stripping at 5 mA cm^−2^. (k) Voltage profiles during CE tests.

To elucidate the molecular basis of interfacial stabilization, we first examined the physicochemical characteristics of the organic component. The calculated dipole moment of PBA (3.5 D), which is substantially higher than that of H_2_O (2.2 D), together with its electrostatic potential (ESP) map showing pronounced charge separation (fig2 and, Figure S10), reveals that PBA carries a strong intrinsic dipole capable of modulating the interfacial electrostatic environment. DFT results further show that PBA forms moderately strong coordination with Zn^2+^ while significantly weakening Zn^2+–^H_2_O interactions (Figure 2b), thereby reducing the affinity of Zn^2+^ for water molecules.

To resolve how PBA couples with the fluorinated interphase at the atomic scale, we evaluated its adsorption behavior on the ZnF_2_ (211) surface. The optimized configuration reveals that PBA binds to the ZnF_2_ surface by a dual N/Br interaction mode, involving N–Zn coordination coupled with Br‐induced σ‐hole polarization, yielding a strong adsorption energy of −1.54 eV (Figure 2c). In contrast, alternative adsorption geometries on ZnF_2_ (211) exhibit significantly weaker binding energies (−0.66 to −0.76 eV; Figure S11), confirming that the N/Br dual‐site configuration is thermodynamically favored. Charge density difference mapping further reveals pronounced electron redistribution at both interaction sites (Figure 2d), demonstrating that the –NH_2_ and –Br groups cooperatively immobilize PBA to form a stable molecular lock. Such dual‐site anchoring electronically integrates PBA into the ZnF_2_ matrix rather than leaving it weakly physisorbed, enabling persistent dipole regulation during cycling. Importantly, once N/Br anchoring saturates the exposed Zn^2+^ sites on the ZnF_2_ surface, water adsorption is strongly suppressed: H_2_O binds ZnF_2_ (211) with −0.77 eV but only −0.05 eV on the PBA‐covered surface (Figure S12). This suppression arises because dual‐site anchoring screens these Lewis‐acidic Zn^2+^ sites on ZnF_2_, preventing favorable H_2_O–Zn^2+^ interactions.

The projected density of states (PDOS) (Figure 2e,f) analysis provides further insight into the bonding nature. Distinct hybridized states between the N*_2p_*/Br*_4p_* orbitals of PBA and the Zn*_3d_* orbitals of ZnF_2_ confirm chemisorption and interfacial orbital coupling. The partial overlap of Br*_4p_* and Zn*_3d_* states is consistent with σ‐hole‐mediated polarization, which electronically reinforces the fluorinated interphase. These electronic features together indicate that PBA–ZnF_2_ coupling and directional dipole alignment cooperatively establish a chemically robust and electronically regulated interphase. Such an interphase is consistent with suppressed water‐induced surface reactions, enhanced Zn^2+^ desolvation, and more homogeneous interfacial electric fields—conditions favorable for dendrite‐free Zn deposition and reduced parasitic reactions.

Building upon this mechanistic understanding, static immersion tests were conducted to evaluate the protective capability of the molecular‐lock SEI. X‐ray diffraction (XRD) analysis shows that the bare Zn electrode surface is almost entirely covered with Zn_4_SO_4_(OH)_6_∙5H_2_O byproducts, whereas negligible byproduct formation is observed on the SEI‐protected Zn foil (Figure 2g). SEM images further show that the SEI‐protected Zn retains a smooth and intact surface morphology after soaking (Figure S13a), while the unprotected Zn electrode develops extensive dendritic structures (Figure S13b). These results verify that the molecular‐lock SEI effectively mitigates electrolyte‐driven corrosion and preserves interfacial integrity.

Electrochemical corrosion analysis further supports this behavior. Tafel polarization reveals markedly reduced corrosion currents in the BDTF‐containing electrolyte (Figure S13c), indicating suppressed corrosion kinetics. Additionally, linear sweep voltammetry (LSV) shows that Zn electrodes with molecular‐lock SEI exhibit more negative HER onset potential (–0.21 V vs Zn^2+^/Zn) compared to the bare Zn anode (0.05 V vs Zn^2+^/Zn) (Figure 2h). in situ pH measurements further reveal the interfacial regulation imparted by the molecular‐lock SEI (Figure 2i and Figure S14a). In the blank electrolyte, the interfacial pH fluctuates strongly (∼4.0–5.1) during cycling, reflecting vigorous proton consumption/production associated with HER. In contrast, the BDTF‐containing electrolyte maintains a narrower pH fluctuation range (∼3.8–4.5), indicating stabilized proton activity and suppressed parasitic reactions. Such suppression of pH oscillation indicates that the SEI limits water access and moderates proton activity, thereby mitigating HER and preventing the interfacial pH from rising.

Having established the interfacial stability, we next assessed the reversibility of Zn plating/stripping in Zn|Cu cells. The Zn|Cu cell with the BDTF‐containing electrolyte exhibits a high initial Coulombic efficiency (CE) of 96.6% and an average CE of 98.7% over the first 10 cycles, indicative of highly reversible Zn plating/stripping. In contrast, Zn|Cu cells in the blank ZnSO_4_ electrolyte show only 63.6% CE in the first cycle and an average CE of 92.1% over the first 10 cycles, consistent with severe parasitic reactions and HER during initial cycling. Furthermore, the Zn anode in the BDTF‐containing electrolyte maintains a stable CE of 99.8% for over 900 cycles, whereas the control cell fails within 300 cycles (Figure 2j,k and Figure S14b). Dynamic electrochemical impedance spectroscopy (EIS) combined with distribution of relaxation time (DRT) analysis further shows that the BDTF‐containing cell rapidly establishes a stable interphase with low interfacial resistance, in contrast to the progressively deteriorating interphase in the blank electrolyte (Figure S15).

**FIGURE 3:**
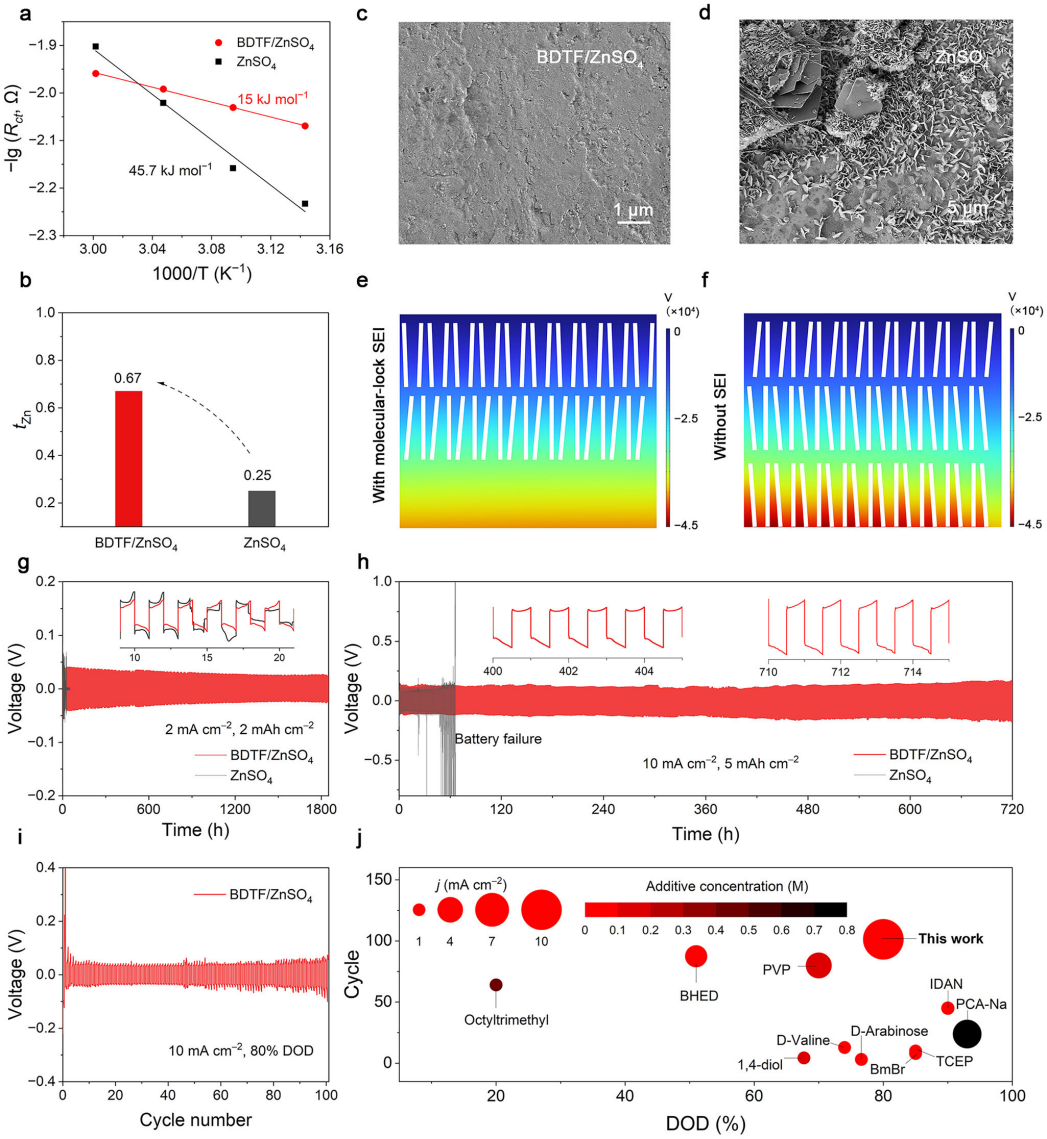
Zn^2^^+^ transport kinetics, interfacial field modulation, and cycling stability enabled by the molecular‐lock SEI. (a) Arrhenius plots of the Zn symmetric cells with different electrolytes. (b) *t*_Zn_​ with different electrolytes. SEM images of the Zn anode after deposition: (c) BDTF‐containing electrolyte; (d) blank electrolyte. Simulated electric field distribution in (e) BDTF‐containing system and (f) ZnSO_4_ system (the thin strip represents glass fiber separator). (g) Long‐term cycling voltage profile at 2 mA cm^−2^ with 2 mAh cm^−2^. (h) Voltage profiles of Zn symmetric cells with/without the BDTF additive (10 mA cm^−2^, 5 mAh cm^−2^). (i) Voltage profile at 10 mA cm^−2^ under 80% DOD in the BDTF‐containing electrolyte. (j) Comparisons of Zn symmetric cells with the BDTF additive and previous literature (based on additive concentration, DOD, current density, and lifespan).

We next examined how the molecular‐lock SEI influences Zn^2+^ desolvation and charge‐transfer kinetics. Zn^2+^ desolvation is usually regarded as the rate‐determining step for Zn deposition, and its activation energy (*E*_a_) can be obtained using the Arrhenius equation (Equation 2):

(2) $$\frac{1}{R_{\mathrm{ct}}}=Ae^{\frac{-E_{a}}{RT}}$$

where *R*_ct_ is the charge‐transfer resistance, *A* is the pre‐exponential factor, *R* is the gas constant, and *T* is the absolute temperature. The BDTF‐containing symmetric cell exhibits a much lower *R*_ct_ than that of the blank electrolyte system over 35–50°C (Figure S16 and Tables S3,S4), confirming accelerated charge‐transfer kinetics. Based on the Arrhenius fitting, the calculated *E*_a_ for the BDTF‐containing symmetric cell is 15.0 kJ mol^−1^, which is approximately 33% of that for the control cell without SEI (45.7 kJ mol^−1^) (Figure 3a), indicating a substantially facilitated Zn^2+^ desolvation process.

To further evaluate the ion transport behavior, the Zn^2+^ transference numbers (*t*_Zn_) were determined using the Bruce–Vincent method [35]. The BDTF‐containing electrolyte exhibits a high *t*_Zn_ of 0.67, far exceeding that of the blank electrolyte (0.25), indicating more efficient Zn^2+^ ion transport and reduced anion competition at the interface (Figure 3b; Figure S17a–d, and Table S5). Cyclic voltammetry (CV) measurements further reveal accelerated redox kinetics in the presence of the molecular‐lock SEI, as evidenced by higher peak current and more symmetric Zn plating/stripping profiles (Figure S17e,f).

SEM images reveal dense and uniform Zn deposition in the BDTF‐containing electrolyte (Figure 3c and Figure S18a), whereas dendritic and loosely packed structures dominate in the additive‐free system (Figure 3d and Figure S18b). Finite‐element simulation further clarifies the mechanism underlying the observed uniform Zn deposition. The introduction of the BDTF‐derived SEI leads to a significantly homogenized electric field distribution at the Zn‐electrolyte interface, effectively mitigating localized field enhancements that typically drive uneven ion flux (Figure 3e). In contrast, the additive‐free system exhibits pronounced electric field concentration at the Zn surface, which promotes tip‐directed Zn growth and dendritic formation (Figure 3f).

To evaluate the rate capability and long‐term stability of the Zn anode, galvanostatic cycling tests were performed on symmetric cells assembled with different electrolytes. The BDTF‐containing symmetric cell exhibits excellent cycling stability, maintaining over 1800 h of stable operation at 2 mA cm^−2^ (2 mAh cm^−2^), which is approximately 15 times longer than that of the control cell without additives (Figure 3g). Moreover, the symmetric cell with BDTF demonstrated superior rate performance and a lower overpotential compared to the additive‐free cell, indicating enhanced Zn plating/stripping kinetics due to the effective suppression of parasitic side reactions (Figure S19).

Even under harsher conditions of 10 mA cm^−2^ and a high areal capacity of 5 mAh cm^−2^, the BDTF‐containing symmetric cell also maintained significantly improved cycling stability, operating for over 700 h without short‐circuit failure (Figure 3h). In contrast, symmetric cells utilizing electrolytes containing Zn(BF_4_)_2_ or Zn electrode modified with a PBA coating rapidly failed due to short‐circuiting, demonstrating the superior ability of the molecular‐lock SEI in suppressing Zn dendrite growth (Figure S20). Furthermore, DOD tests revealed that the BDTF‐modified cell could sustain stable cycling for more than 100 cycles under 80% DOD at a current density of 10 mA cm^−2^ (Figure 3i). Compared with previously reported additive or SEI strategies that typically achieve high capacities at low current densities (1 mA cm^−2^) or limited cycle life (<60 cycles) at high rates—and often rely on high concentrations of electrolyte additives—our system achieves higher DOD (80%) and longer cycling lifespan (100 cycles), even at a higher current density of 10 mA cm^−2^ despite the extremely low concentration (1 mM) of the BDTF additive (Figure 3j and Table S6) [6, 24, 27, 36, 37, 38, 39, 40, 41, 42].

**FIGURE 4:**
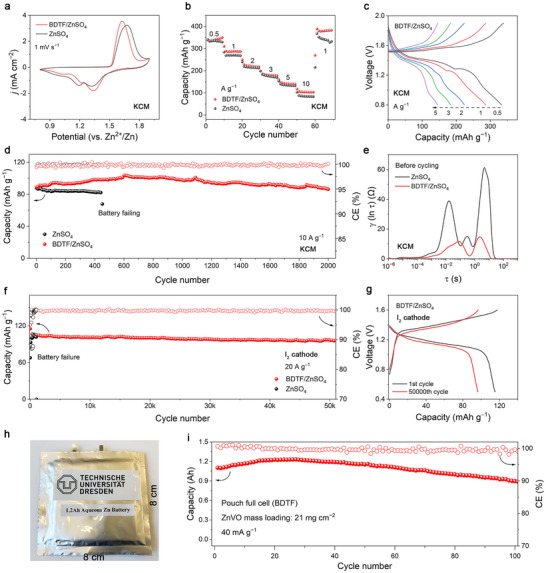
A broadly applicable, molecular‐lock SEI for diverse Zn‐based battery chemistries. (a) CV curves of Zn|KCM full cells with/without the BDTF additive. (b) Rate capability measurements from 0.5 to 10 A g^−1^. (c) Voltage profiles across varying rates of the BDTF‐containing full cell. (d) Cycling performance and (e) DRT spectra of the Zn|KCM full cells with/without the BDTF additive. (f) Cycling stability of Zn|I_2_ cells at an exceptionally high current density of 20 A g^−1^. (g) Charge–discharge curves of Zn|I_2_ cells with the BDTF additive during the first and 50000th cycles. (h) Optical image of the assembled pouch full cell. (i) Cycling stability of the Ah‐level Zn|ZnVO pouch cell with BDTF‐containing electrolyte.

To demonstrate the practical applicability of the BDTF additive, Zn metal anodes were coupled with K, Cu‐codoped MnO_2_ (KCM) cathodes in coin cell configurations (Figure S21). Cyclic voltammetry reveals higher redox peak currents and lower overpotentials in the BDTF‐containing cells, indicating accelerated redox kinetics (Figure 4a). The BDTF‐enabled Zn|KCM full cell delivers comparable specific capacities of 340 mAh g^−1^ at 0.5 A g^−1^ and 153.5 mAh g^−1^ at 5 A g^−1^ (based on the mass of active KCM) relative to control cells (Figure 4b,c and Figure S22). Furthermore, upon reducing the current back to 0.5 A g^−1^, the BDTF‐containing full cell recovers a high specific capacity of 378.5 mAh g^−1^, demonstrating excellent rate capability. Notably, the BDTF‐free full cell suffered from early short‐circuit failure, whereas the BDTF‐containing full cell maintained excellent capacity retention of 99.3% after 2000 cycles, with an average CE approaching 100% (Figure 4d). Importantly, this long‐term stability outperforms previously reported strategies involving artificial SEI layers, molecular additives, or gel‐based electrolytes (Table S7), demonstrating the exceptional durability enabled by the programmed molecular‐lock SEI.

DRT analysis was used to resolve the interfacial reaction processes contributing to full‐cell polarization. Compared with the additive‐free system, the BDTF‐containing full cell shows significantly suppressed relaxation peaks in both the charge‐transfer region (∼10^−2^–10^0^ s) and the intermediate‐frequency regime (∼1 s) (Figure 4e). The reduction of the charge‐transfer peak indicates lower interfacial impedance, while the weakened intermediate‐frequency peak reflects significantly mitigated side‐reaction kinetics due to the programmed molecular‐lock SEI.

To assess the general applicability of the molecular‐lock SEI, the strategy was further applied to Zn–I_2_ batteries, which operate through a polyiodide conversion mechanism. Benefiting from the programmed organic‐lock SEI, Zn|I_2_ cells deliver exceptional durability for 50,000 cycles at a high current density of 20 A g^−1^, retaining 84% of their initial capacity with an ultralow per‐cycle decay rate of only 0.00323‰ (Figure 4f,g and Figure S23). In contrast, the additive‐free Zn|I_2_ cells exhibit pronounced capacity fluctuations and rapidly undergo short‐circuit failure after 1000 cycles, illustrating the severity of interfacial instability in the absence of protective SEI.

Post‐cycling analysis of the Zn anodes clarifies the origin of this difference. SEM reveals that the additive‐free electrolyte induces rough, dendritic deposits, whereas the BDTF‐containing electrolyte yields a smooth and compact Zn surface. Furthermore, EDS mapping and XPS analysis confirm that the key SEI constituents—N, F, and Br—remain stable after 500 cycles (Figure S24), demonstrating the chemical robustness of the SEI against polyiodide‐induced degradation. These results confirm that the molecular‐lock SEI provides robust and broadly applicable interfacial protection, maintaining stability even in the highly reactive polyiodide environment of Zn–I_2_ batteries.

To evaluate the feasibility of scaling this strategy toward practical devices, an 8 cm × 8 cm multi‐layer pouch cell was assembled using a Zn_x_V_2_O_5_·nH_2_O (ZnVO) cathode with ultrahigh loading of 21 mg cm^−2^ and tested for cycling stability (Figure 4h,i). The BDTF‐containing pouch cell achieves the highest capacity of 1.2 Ah and retains 81% of its capacity after 100 cycles at a current density of 40 mA g^−1^ (based on the active mass of ZnVO) (Figure S25). This performance exceeds that of previously reported Zn pouch cells that employed strategies such as electrolyte additives and gel electrolytes, in combination with various cathode materials (e.g., MnO_2_ and ZnVO) (Table S8) [43, 44, 45, 46, 47, 48, 49, 50, 51, 52] The 1.2 Ah aqueous Zn pouch cell can also steadily power a commercial energy‐saving lamp, demonstrating its operational practicality (Figure S26). Based on the overall cell configuration, the BDTF‐enabled pouch cell exhibits a gravimetric energy density of approximately 37 Wh kg^−1^ and a volumetric energy density of 29.6 Wh L^−1^ (Table S9). These values, achieved with only trace additive incorporation in a conventional aqueous electrolyte platform, underscore the scalability and real‐device relevance of the molecular‐lock SEI strategy.

## 3 Conclusion

In summary, we report an electron‐induced molecular programming strategy that constructs a Zn^2+^‐selective molecular‐lock interphase using only 1 mm BDTF. Electrochemical reduction of the diazonium precursor and simultaneous fluorination generate PBA species that anchor onto ZnF_2_ nanodomains, forming an ultrathin, compositionally graded, and field‐regulating interphase. This programmed molecular‐lock architecture embeds directional dipoles, stabilizes ZnF_2_, guides Zn^2+^ flux, and repels interfacial water—capabilities unattainable for conventional SEIs formed through stochastic reactions. Benefiting from this molecularly defined interphase, Zn anodes deliver a CE of 99.8% and sustain stable plating/stripping under 80% DOD at 10 mA cm^−2^. The strategy further enables long‐life full cells, including 2000‐cycle stability in Mn‐based systems and 50,000‐cycle durability in Zn–I_2_ batteries. Moreover, an 8 cm × 8 cm Zn|ZnVO pouch cell with 21 mg cm^−2^ loading achieves 1.2 Ah with 81% retention using only a trace additive. Overall, this work establishes a programmable molecular‐level framework for interfacial regulation and provides a new paradigm for interfacial chemistry in aqueous and multivalent metal batteries.

## Conflicts of Interest

The authors declare no conflicts of interest.

## Supporting information




**Supporting File**: adma72891‐sup‐0001‐SuppMat.docx.

## Data Availability

The data that support the findings of this study are available from the corresponding author upon reasonable request.
