# Supplementary material for: Electron‐Induced Molecular Programming Drives Interfacial Chemistry for Ah‐Level Zinc Batteries
Source: Adv Mater. 2026 Mar 24;38(23):e72891. doi: 10.1002/adma.72891 (PMC13103630; doi:10.1002/adma.72891)
Supplement: Supplementary file 1 — Supporting File: adma72891‐sup‐0001‐SuppMat.docx. [file ADMA-38-e72891-s001.docx]

**Supporting Information:**

**Electron-Induced Molecular Programming Drives Interfacial Chemistry for** **Ah-****Level Zinc Batteries**

*Feifei Wang**^1,2^, Yuhang Zhuang^2^, Jiwei Shi^3^, Haojie Zhang^1^, Peng Zhang^1,2^, Songshan Bi^2^, Hyejung Yang^2^, Wenqiang Yang^2^, Stuart S.P. Parkin^1^, Chunpeng Yang^3^, Quan-Hong Yang^3^, Ali Shaygan Nia^1,2^** *& Xinliang Feng^1,2^**

^1^ Max Planck Institute for Microstructure Physics, D-06120 Halle (Saale), Germany

^2^ Center for Advancing Electronics Dresden (cfaed) and Faculty of Chemistry and Food Chemistry, Technische Universität Dresden, 01062 Dresden, Germany

^3^ Nanoyang Group, Tianjin Key Laboratory of Advanced Carbon and Electrochemical Energy Storage, School of Chemical Engineering and Technology, and National Industry-Education Integration Platform of Energy Storage, Tianjin University, 300072 Tianjin, China

*E-mail: ashaygan@mpi-halle.mpg.de, xinliang.feng@mpi-halle.mpg.de

*^#^*Feifei Wang and Yuhang Zhuang contribute equally to this work.

**Experimental sections**

**Preparation of the BDTF/ZnSO_4_ electrolyte**

A 2.0 M ZnSO_4_ aqueous solution was first prepared by dissolving ZnSO_4_·7H_2_O in deionized water. Subsequently, 4-bromobenzenediazonium tetrafluoroborate (BDTF) was directly dissolved into the ZnSO_4_ solution to obtain a final concentration of 1 mM. The resulting electrolyte was clear and homogeneous, exhibiting good stability under ambient conditions without visible precipitation or discolouration for at least 10 days (Figure S1). The electrolyte containing 1 mM BDTF maintains a pH comparable to that of the blank ZnSO_4_ solution and exhibits the most favorable interfacial behavior among the tested concentrations. Higher additive concentrations do not further improve Zn reversibility but instead increase interfacial resistance (Figure S2, S3). Therefore, 1 mM was selected for subsequent electrochemical investigations.

**Preparation of the KCM cathode**

KCM nanomaterial was synthesized via a simple coprecipitation method based on previously reported procedures ^[1]^ with a key modification of replacing sodium hydroxide (NaOH) with potassium hydroxide (KOH) as the precipitating agent.

**Preparation of the ZnVO cathode**

The ZnVO powder was synthesized following a previously reported method with slight modifications. Specifically, 0.2728 g of V_2_O_5_ and 0.2140 g of zinc acetate Zn(CH_3_COO)_2_ were dissolved in 35 mL of deionized water under stirring. Subsequently, 5 mL of acetone and 2 mL of 10% nitric acid were added to the above solution. After ultrasonication for 5 minutes to ensure thorough mixing, the resulting mixture was transferred into a 60 mL Teflon-lined stainless-steel autoclave (Anhui Kemi Machinery Technology Co., Ltd) and heated at 180 °C for 24 hours. After the autoclave naturally cooled to room temperature, the precipitate was collected by centrifugation, washed several times with deionized water and ethanol, and then dried under vacuum at 80 °C for 12 hours to obtain the final ZnVO powder.

**Preparation of the I_2_ cathode**

The cathode slurry was prepared by dispersing Ketjen black (KB) and PTFE binder (Canrd Technology Co., Ltd.) in a mass ratio of 9:1 in ethanol, followed by thorough mixing to obtain a homogeneous paste. The slurry was uniformly coated onto carbon cloth and subsequently dried under vacuum to yield the KB-based electrode. The catholyte was formulated by dissolving I_2_ (0.1 M) and LiI (1 M) in deionized water, while the anolyte consisted of an aqueous 2 M ZnSO_4_ solution. CR2032-type Zn–I_2_ coin cells were assembled using Zn foil as the anode, with 10 μL of catholyte along with 50 μL of anolyte. The areal loading of active iodine species was approximately 2 mg cm^−2^.

**Material characterization**

XRD analysis (Bruker D8, Cu Kα, λ = 0.154 nm, 25 °C) was performed on Zn foils that had been pre-cycled 7 times in the BDTF/ZnSO₄ electrolyte before the corrosion experiments, ensuring that the SEI layer examined corresponded to the electrochemically formed surface rather than a freshly polished Zn substrate. Microstructural and elemental analyses were performed using a Carl Zeiss Gemini 500 field-emission scanning electron microscope (SEM) equipped with an Oxford X-max N 150 energy-dispersive X-ray spectrometer (EDS). For cross-sectional imaging, the electrodes were immersed in liquid nitrogen and fractured to expose the internal interface. The fractured samples were subsequently examined by SEM under low accelerating voltage to minimize beam-induced charging effects. Transmission electron microscopy (TEM, JEM-3100F) was employed to further investigate the detailed morphology and lattice structure. For TEM characterization, the SEI samples were prepared directly on a 3 mm diameter Cu mesh. Zn was electrodeposited onto the Cu mesh at a current density of 0.5 mA cm^−2^ with a total deposition capacity of 5 mAh. The porous structure of the Cu mesh ensured that Zn was selectively deposited on the conductive areas, leading to a reduced effective deposition area. The resulting Zn-coated Cu mesh was then assembled into CR2032 coin cells and subjected to galvanostatic cycling at 0.5 mA cm^−2^ to form the SEI layer. After cycling, the Cu mesh with the SEI/Zn was retrieved, rinsed with deionized water to remove residual electrolyte, and subsequently dried under vacuum before TEM analysis. Structural and compositional analyses were conducted using X-ray photoelectron spectroscopy (XPS, PHI-5702) with a monochromatic Al Kα X-ray source (1486.6 eV), while Raman spectra were recorded using a WITec confocal Raman microscope equipped with a 532 nm excitation laser. For mass spectrometry, after three cycles, the Zn foil was retrieved, gently rinsed with deionized water, and immersed in 1 mL of acetonitrile for 12 h to extract the organic SEI components. The supernatant was then subjected to mass spectrometry analysis. The measurements were performed using a Waters Xevo G2-XS QTOF mass spectrometer equipped with an electrospray ionization (ESI) source, operated in positive ion mode. The spectra were evaluated to identify *p*-bromoaniline (PBA), characterized by the isotopic molecular ion doublet at m/z 172/174 (1:1, ^79^Br/^81^Br) and the diagnostic fragment at m/z 93, as well as related intermediates.

**Assembly of batteries**

2032-type coin cells were assembled with two identical bare Zn foils (*d* =10 mm, thickness of 100 μm). A glass fiber membrane (GF/D) was used as the separator. 2 M ZnSO_4_ aqueous solutions with or without 1 mM BDTF were used as electrolytes, respectively. For coin-type full cells, the cathode was prepared by casting a slurry composed of KCM powder (70 wt%), Super P (20 wt%), and PVDF (10 wt%) onto a carbon cloth, with an active material loading of 1.5 mg cm^−2^.

**Assembly of a pouch cell**

The Ah-scale pouch full cell was assembled using a cathode prepared by casting a slurry of ZnVO, Super P, and PVDF (weight ratio 7:2:1) onto 5 pieces of carbon cloth (8 × 8 cm^2^), followed by vacuum drying. The areal mass loading of ZnVO was controlled at approximately 21 mg cm^−2^. A bacterial cellulose membrane was employed as the separator. Before cell operation, 2.5 mL of BDTF/ZnSO_4_ aqueous electrolyte was injected. A titanium foil strip was connected to the current collector to serve as the external tab. After electrode stacking and electrolyte injection, the entire cell was sealed in an aluminium–plastic laminate pouch.

**Electrochemical measurements**

Galvanostatic charge and discharge measurements were applied with a Land battery test system (LAND CT2001A). LSV, EIS, and CV tests were performed on a CHI760E electrochemical workstation. For the HER test, LSV was conducted in 1.0 M Na_2_SO_4_ aqueous electrolyte with or without BDTF additives, using a potential window from 0 to −0.9 V (*vs.* Zn^2+^/Zn) at a scan rate of 5 mV s ^−1^. Tafel polarization curves were recorded at 1 mV s ^−1^ in the potential range of –0.3 to +0.3 V (vs. Zn^2+^/Zn). EIS measurements were recorded in the frequency range of 0.1–10^5^ Hz with an AC perturbation amplitude of 5 mV. Before EIS, Tafel, and LSV measurements, the electrodes were preconditioned by 7 galvanostatic cycles in the BDTF/ZnSO_4_ electrolyte to stabilize the interfacial state.

**Finite-element simulation**

In this study, a two-dimensional steady-state electric field model was developed to simulate ion transport and potential distribution in a ZnSO_4_ electrolyte system. The “Conductive Media” physics interface was employed, with a constant current applied at the terminal boundary and a grounded boundary condition set elsewhere. A stationary solver with a fully coupled configuration and the default direct linear solver was used to compute the potential distribution. All simulations were performed under default tolerances. The results were used to assess how additive structures modulate the local electric field.

The core governing equation used in the simulation is the steady-state conductive media equation:

$$\nabla\cdot J=0$$

$$J=\sigma E=-\sigma\nabla V$$

Where *J* is the current density, 𝜎 is the electrical conductivity of the medium, 𝐸 is the electric field, and 𝑉 is the electric potential.

**DFT calculations**

The HOMO and LUMO energy levels of different additives were calculated using the B3LYP functional within the Dmol^3^ module of Materials Studio. The double numerical basis set with polarization functions (DNP 3.5) and an orbital cutoff of 4.4 Å were employed. Geometry optimizations were performed in the gas phase without symmetry constraints, implicit solvation models (e.g., COSMO), or dispersion corrections (e.g., DFT-D). The convergence criteria for geometry optimization were set to 1.0 × 10^−5^ hartree for energy, 0.002 hartree/Å for maximum force, and 0.005 Å for displacement, with a maximum of 50 optimization steps and a step size limit of 0.3 Å. The SCF convergence threshold was set to 1.0 × 10^−6^ hartree, with a smearing width of 0.005 hartree applied to accelerate convergence. Unless otherwise noted, molecules were treated as neutral (charge = 0). For the diazonium cation derived from BDTF, a +1 charge state was used. The formal spin configuration was used as the initial guess. HOMO and LUMO levels were extracted from the converged wavefunction and used to assess the electronic structure and charge transfer characteristics of the additives. Adsorption energy calculations were performed using the Vienna Ab Initio Simulation Package (VASP) with the generalized gradient approximation (GGA) method.^[2–4]^ Electron-ion interactions were treated by the projector augmented wave (PAW) method, and the Perdew-Burke-Ernzerhof (PBE) functional was employed for describing the exchange and correlation energies,^[5]^ and the energy cutoff was set at 400 eV. The Brillouin zone was sampled by a Γ-centered k-point mesh of (2 × 2 × 1). The energy convergence criterion between the two electronic steps was set to 10^−4^ eV, and the system structures before and after adsorption were optimized until the Hellmann-Feynman force of each atom reached below 0.01 and 0.05 eV Å^−1^, respectively.


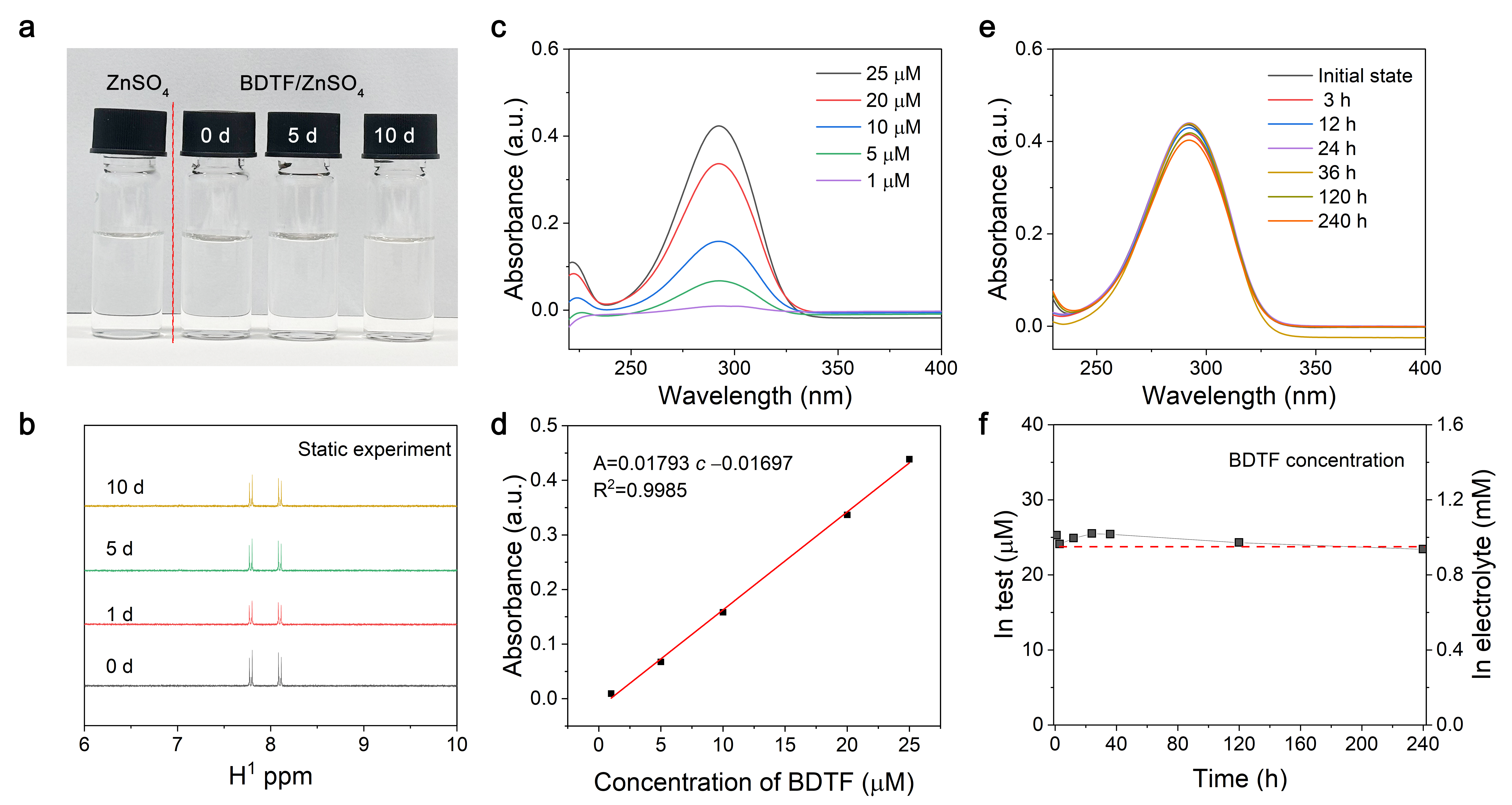


Figure S1. (a) Optical photographs of ZnSO_4_ and BDTF/ZnSO_4_ solutions after different storage times (0, 5, and 10 days). (b) Time-resolved ^1^H NMR spectra of the BDTF/ZnSO_4_ electrolyte over 10 days. (c) UV–vis absorption spectra of BDTF solutions with different concentrations (0.5–25 μM) prepared in 2 M ZnSO_4_ electrolyte. (d) Corresponding calibration curve of absorbance at ~295 nm versus BDTF concentration, showing a linear correlation. (e) UV–vis absorption spectra of the BDTF/ZnSO_4_ electrolyte recorded at various time points from 0 to 240 h. (f) Time-dependent BDTF concentration quantified by UV–vis spectroscopy.

The BDTF/ZnSO_4_ electrolyte remained colorless and transparent for 10 days without any visible precipitation or gas evolution (Figure S1a). The ^1^H NMR spectra of the freshly prepared BDTF/ZnSO_4_ solution (D_2_O, RT) display two characteristic AA′BB′ aromatic doublets at 7.79–7.82 and 8.10–8.13 ppm, corresponding to the four protons of the para-substituted benzene ring. The downfield resonance at ~8.1 ppm is assigned to protons ortho to the electron-withdrawing diazonium group, whereas the 7.8 ppm signal originates from protons adjacent to the bromo substituent (Figure S1b). These chemical shifts are fully consistent with the structure of the *p*-bromobenzenediazonium cation. The persistence of these diagnostic resonances confirms the presence of intact *p*-bromobenzenediazonium species in the electrolyte during the static stability test.

Furthermore, UV–vis spectroscopy corroborates the excellent stability of the BDTF additive. The absorption spectra of BDTF/ZnSO_4_ electrolytes with varying concentrations (Figure S1c,d) exhibit a clear linear relationship between absorbance and concentration, validating quantitative analysis. Time-dependent UV–vis measurements of the same electrolyte (Figure S1e) reveal negligible changes in absorption intensity or peak position during 240 h of storage. The corresponding concentration plot (Figure S1f) confirms a constant BDTF level over time, indicating that the additive remains chemically stable and does not undergo spontaneous hydrolysis or decomposition in the absence of electrochemical polarization.


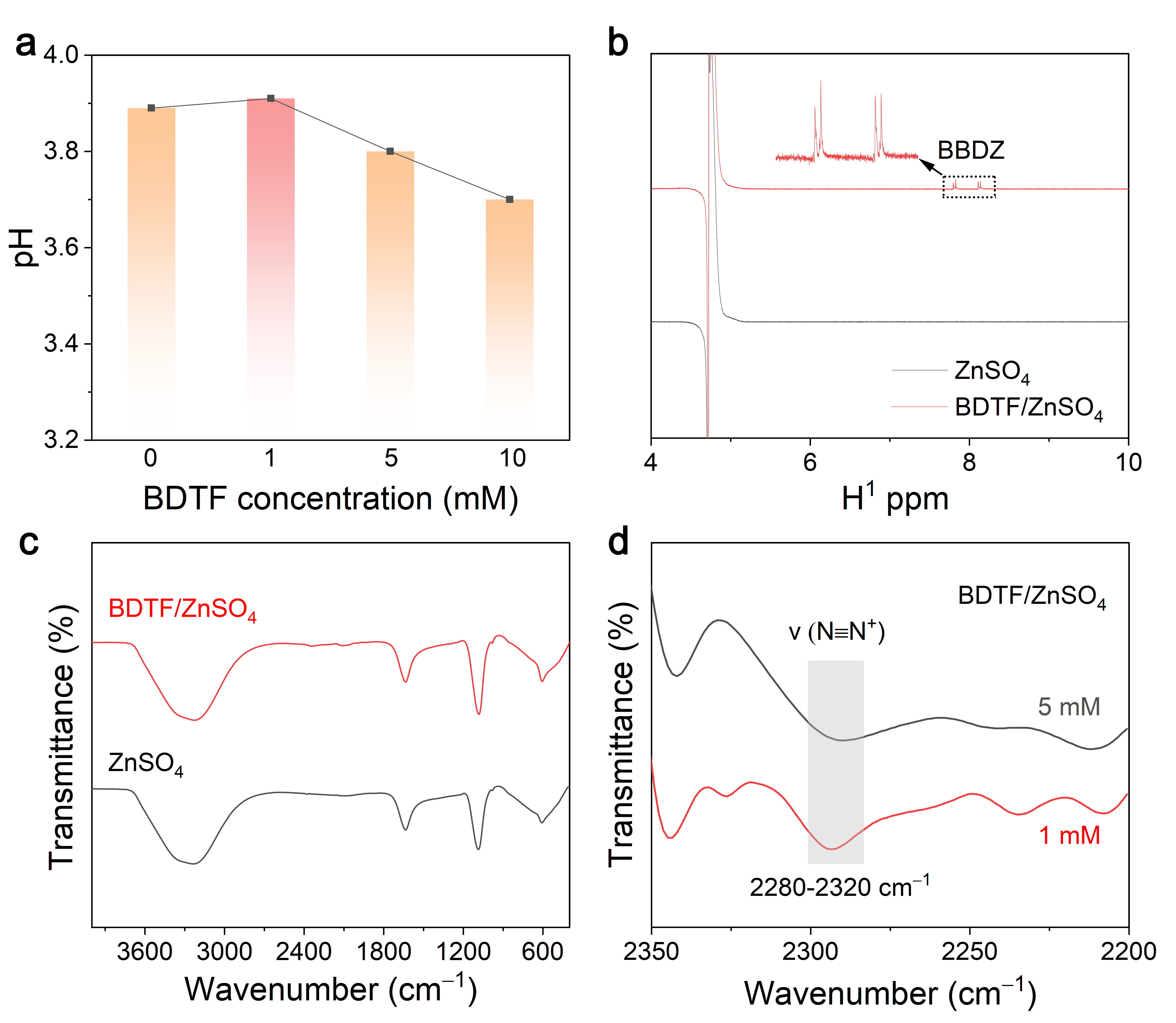


Figure S2. Physicochemical characterization of BDTF-containing ZnSO_4_ electrolytes. **(a)** pH values of ZnSO_4_ with different BDTF concentrations. (b) ^1^H NMR spectra of the BDTF/ZnSO_4_ electrolyte**. (c)** ATR-FTIR spectra of different electrolytes. **(d)** Enlarged FTIR region highlighting the –N≡N^+^ stretching band in 1 mM and 5 mM BDTF/ZnSO_4_ electrolytes.

The ^1^H NMR spectra of the BDTF/ZnSO_4_ electrolyte (Figure S2b) retain the characteristic aromatic resonances of the p-bromobenzenediazonium species without the emergence of new peaks, indicating good chemical stability of BDTF in the bulk electrolyte under open-circuit conditions. Consistently, ATR-FTIR spectra (Figure S2c,d) show negligible changes in the O–H stretching/bending features of coordinated water after BDTF addition, suggesting that the bulk Zn^2+^ solvation structure remains essentially unchanged. Therefore, the interfacial evolution observed during cycling primarily originates from electrochemical transformation of BDTF at the Zn surface rather than bulk solvation restructuring.


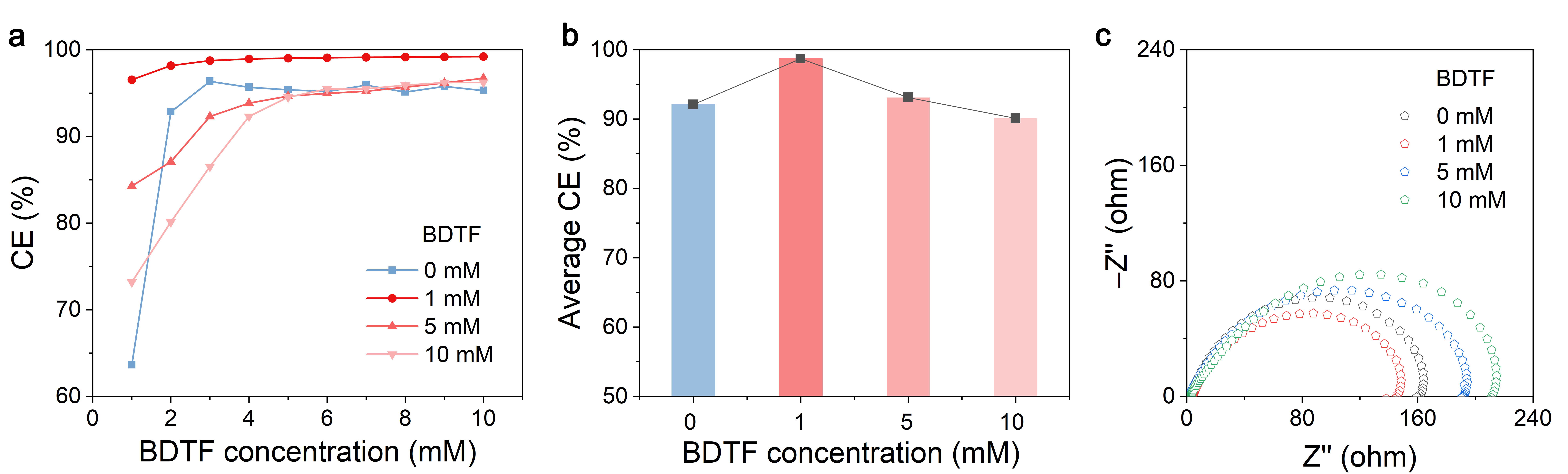


Figure S3. **Effect of BDTF concentration on Zn plating/stripping behavior and interfacial resistance.** (a) CE curves at different BDTF concentrations. (b) Average CE over the initial 10 cycles as a function of BDTF concentration. (c) Nyquist plots obtained from EIS measurements at different BDTF concentrations.

As shown in Figure S3a, the electrolyte containing 1 mM BDTF exhibits the highest initial CE. In contrast, further increasing the additive concentration to 5 and 10 mM does not improve reversibility and instead results in slightly lower CE values during early cycles. The average CE over the first 10 cycles (Figure S3b) confirms this trend, where 1 mM BDTF delivers the optimal reversibility.

Electrochemical impedance spectroscopy (Figure S3c) further reveals that higher BDTF concentrations result in increased charge-transfer resistance (*R*ct), indicating the formation of a thicker, more resistive interphase. These results demonstrate that excessive additive concentration induces overgrowth of the interphase, which hinders interfacial kinetics rather than improving stability.

Collectively, these findings confirm that 1 mM BDTF represents an optimized concentration that balances effective interfacial programming with minimal kinetic penalty. Therefore, 1 mM BDTF was selected for all subsequent electrochemical investigations.


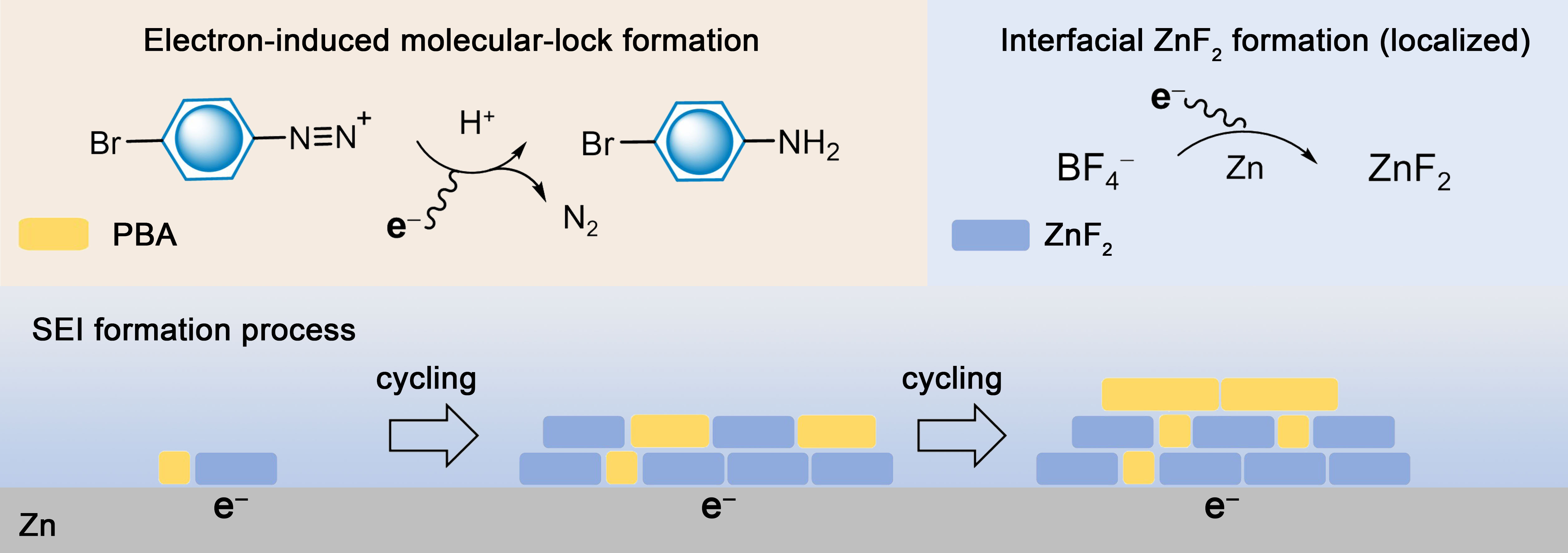


Figure S4. Schematic illustration of the electron-induced interfacial programming mechanism for molecular-lock SEI formation.

To clarify the *in situ* formation sequence and underlying reaction mechanism, we provide a stepwise schematic summary of the interfacial evolution process. The formation of the molecular-lock SEI is initiated by preferential reduction of the diazonium species, which occurs at a higher potential than water reduction. The generated PBA molecules remain confined at the Zn surface and interact with concurrently formed ZnF_2_ species arising from localized BF_4_⁻ activation. Rather than forming independent organic and inorganic layers, these species assemble cooperatively through defined interfacial bonding. The embedded dipolar organic species further reorganize the interfacial electrostatic environment, contributing to ZnF_2_ stabilization, water exclusion, and facilitated Zn^2+^ transport. This schematic integrates the spectroscopic, microscopic, and theoretical evidence presented in the main text into a unified mechanistic framework.


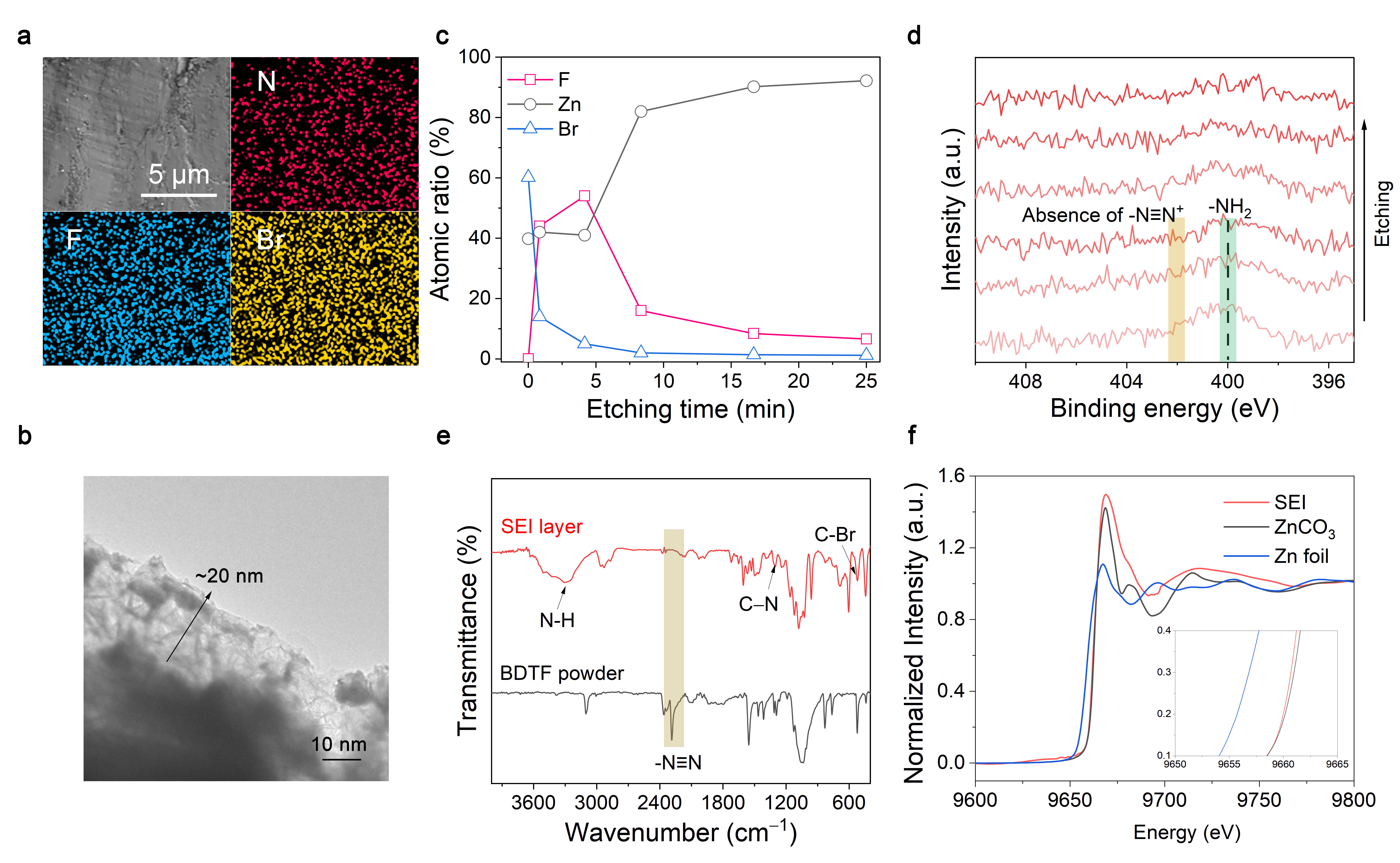


Figure S5. (a) Elemental mapping of Zn, F, and Br elements. (b) TEM image of the hybrid SEI layer on the Zn electrode. (c) Calculated ratios of Zn 2p, F 1s, and Br 3d. (d) Depth-resolved XPS profiles of N 1s. (e) ATR-FTIR spectra of the hybrid SEI layer on the Zn electrode. (f) Zn K-edge XANES spectra of SEI/Zn, ZnCO_3_, and Zn foil.


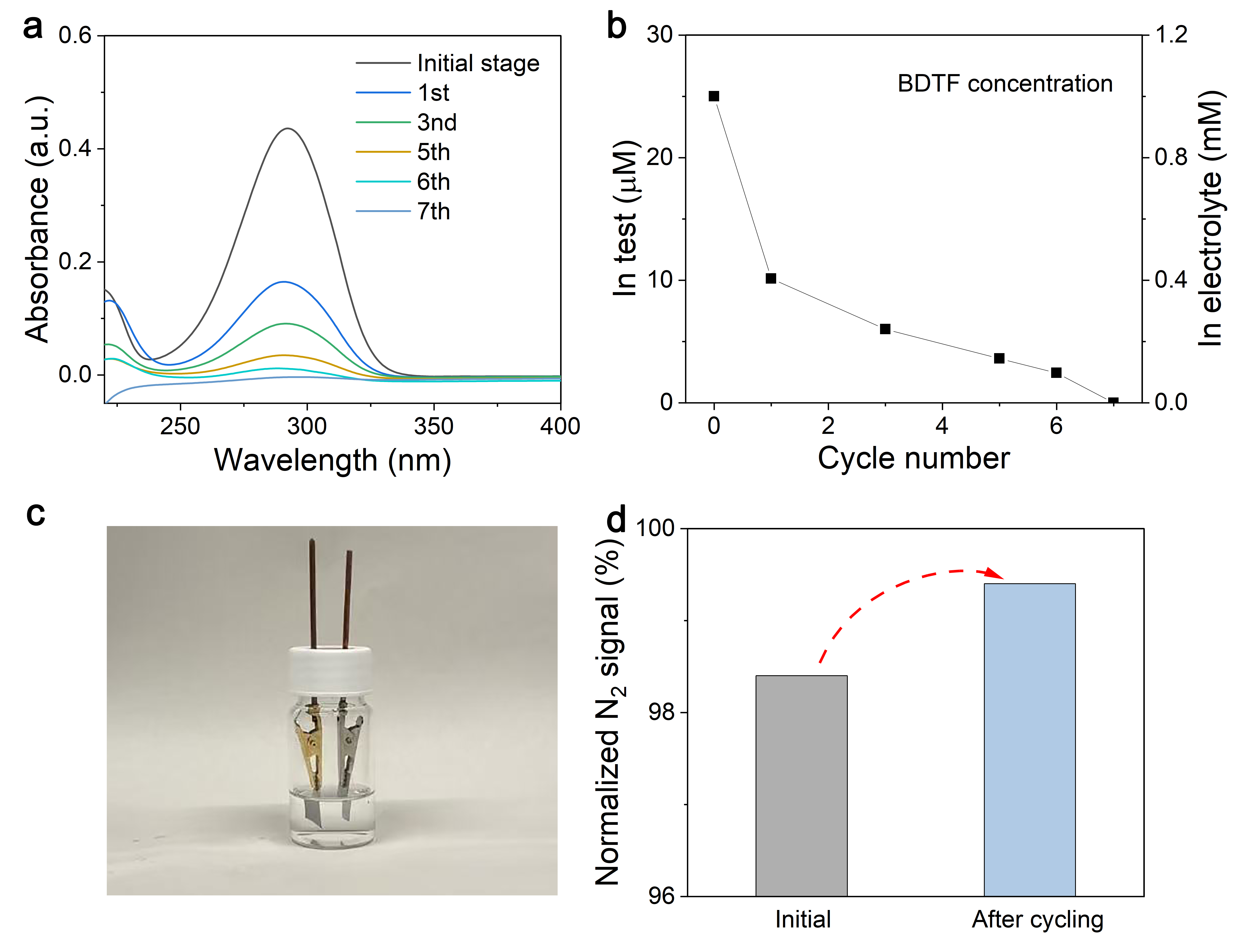


Figure S6. (a) UV–Vis spectra of the electrolyte sampled at different cycling stages. (b) Calculated BDTF concentrations as a function of cycle number. (c) GC–TCD measurement device of headspace gases before and after Zn symmetric cell cycling. (d) Comparison of normalized N_2_ signal before and after cycling, indicating N_2_ generation from diazonium reduction.


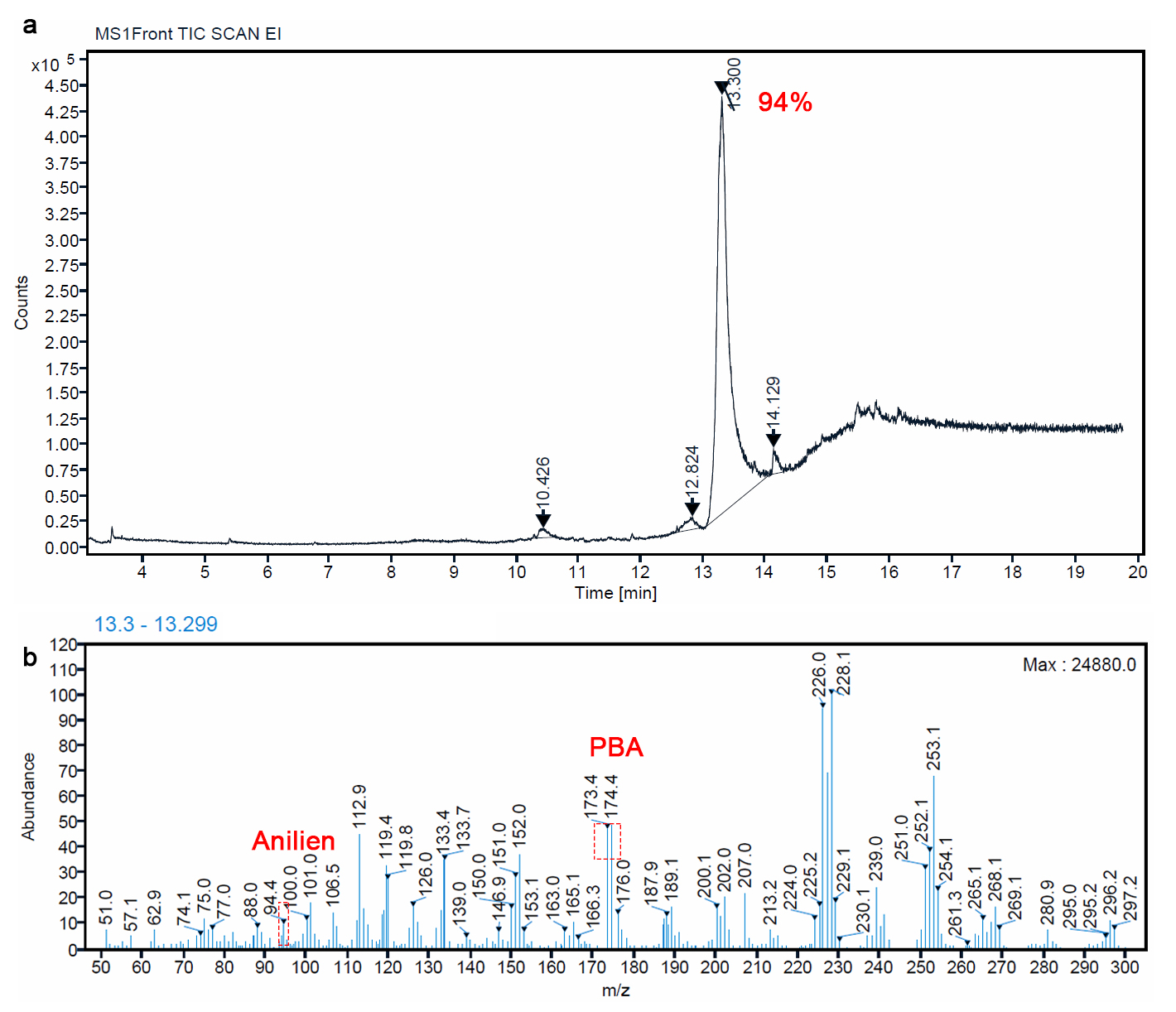


Figure S7. (a) Total ion chromatogram of the SEI extracts, showing a dominant peak at ~13.5 min corresponding to the main organic product (~94% relative intensity). (b) Mass spectrum of the major product (13.3 min), indicating the formation of p-bromoaniline (PBA, m/z = 172.4) and aniline-related fragments, confirming the electron-induced transformation of BDTF.

Mass spectrometry analysis was conducted on the organic components extracted from the SEI formed in the BDTF-containing electrolyte (Figure S7). The total ion chromatogram exhibits a dominant peak at approximately 13.3 min, accounting for ~94% of the total ion intensity. The corresponding mass spectrum reveals characteristic fragment ions at m/z = 172.4, 157.4, and 144.3, which are assigned to PBA and its aniline-related derivatives. These results provide direct molecular-level evidence that the BDTF additive undergoes electrochemical reduction to form PBA during cycling.


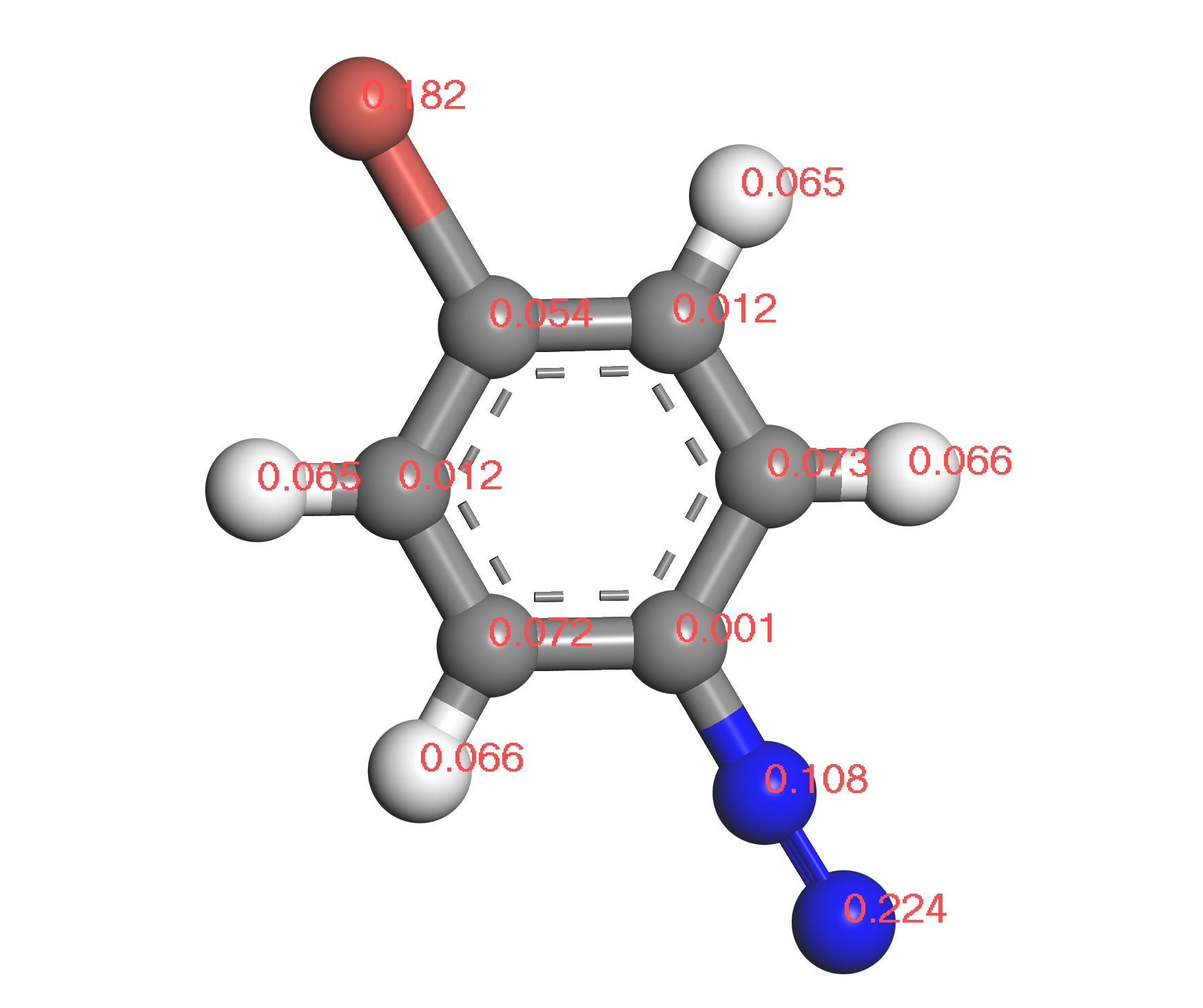


Figure S8. Fukui function *f ^−^* distribution of BBDZ, indicating the most probable site for electron uptake (reduction).


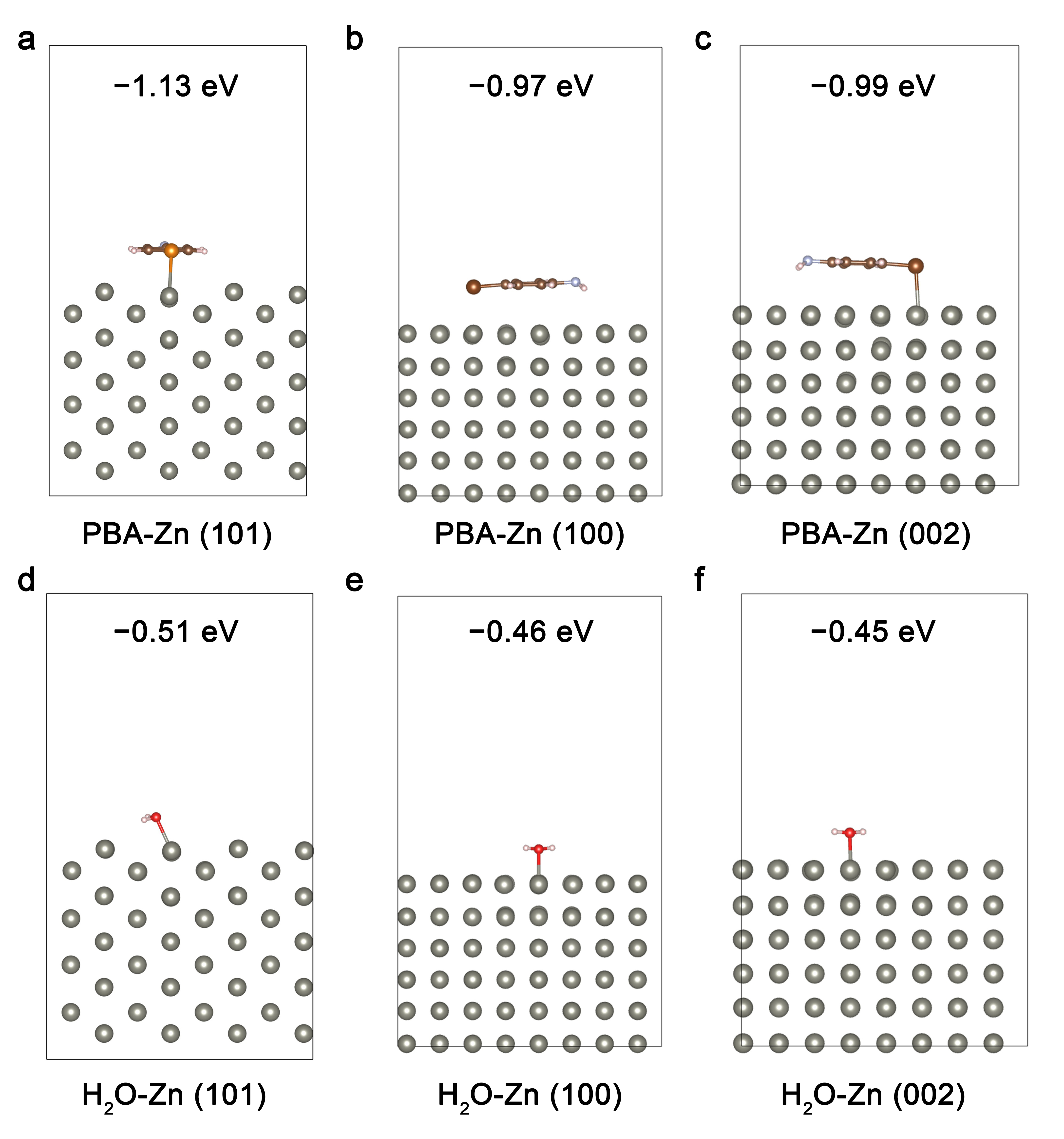


Figure S9. Adsorption configurations of H_2_O and PBA molecules on (a, d) Zn (101), (b, e) Zn (100), and (c, f) Zn (002) facets, with the corresponding adsorption energies labeled above each configuration.


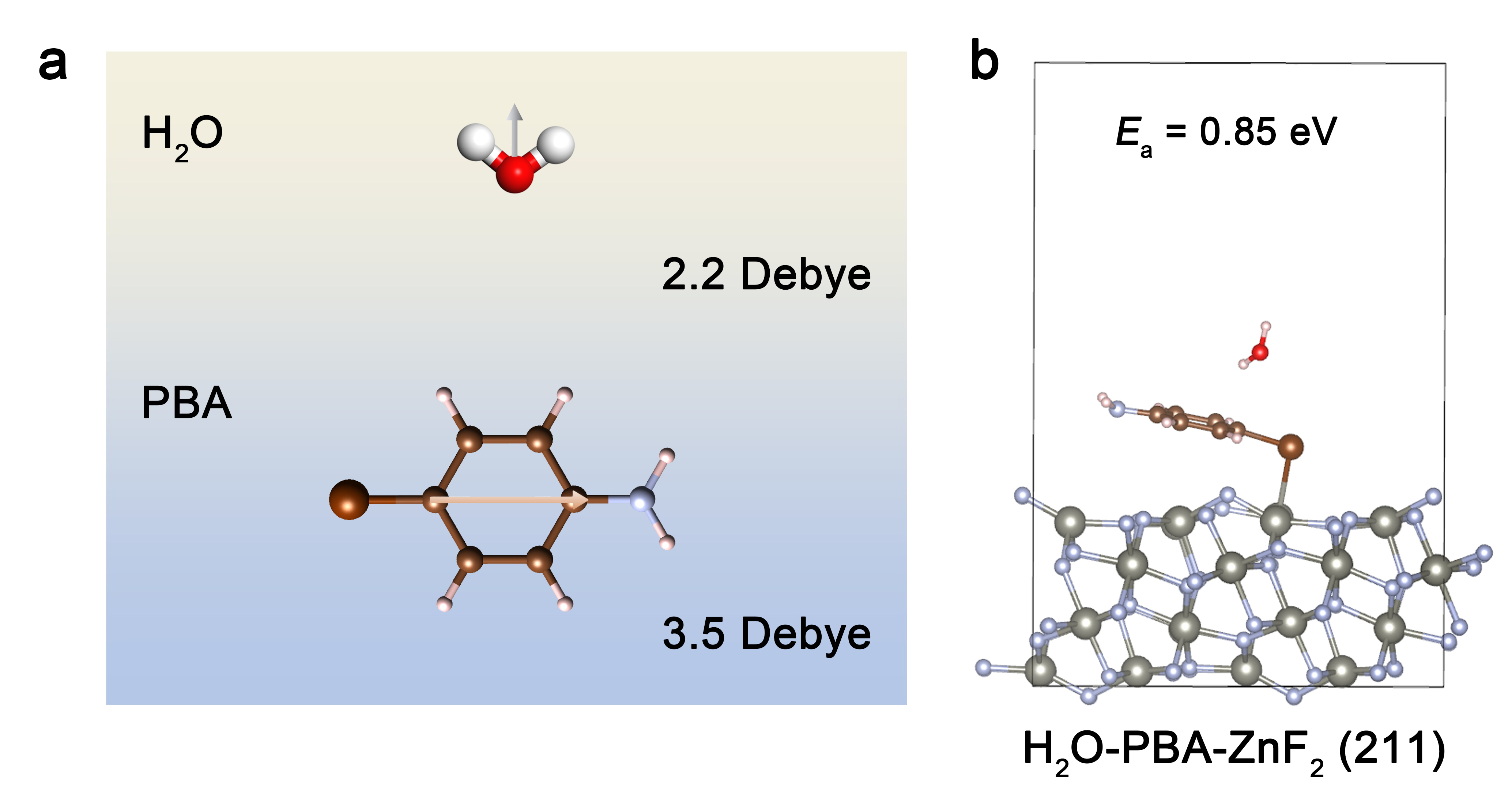


Figure S10. Dipole moment comparison between H_2_O and PBA.





Figure S11. Representative PBA adsorption geometries on the ZnF_2_ (211) surface. (a–c) Three alternative binding configurations with adsorption energies of –0.66, –0.75, and –0.76 eV, respectively.

Top and side views are shown for each structure. All configurations exhibit exothermic adsorption, confirming the intrinsic affinity of PBA for the fluorinated surface. Although multiple metastable geometries exist, their adsorption energies are significantly weaker than that of the most stable dual N/Br anchoring mode (–1.54 eV), validating its thermodynamic preference and use as the representative model in Figure 2.


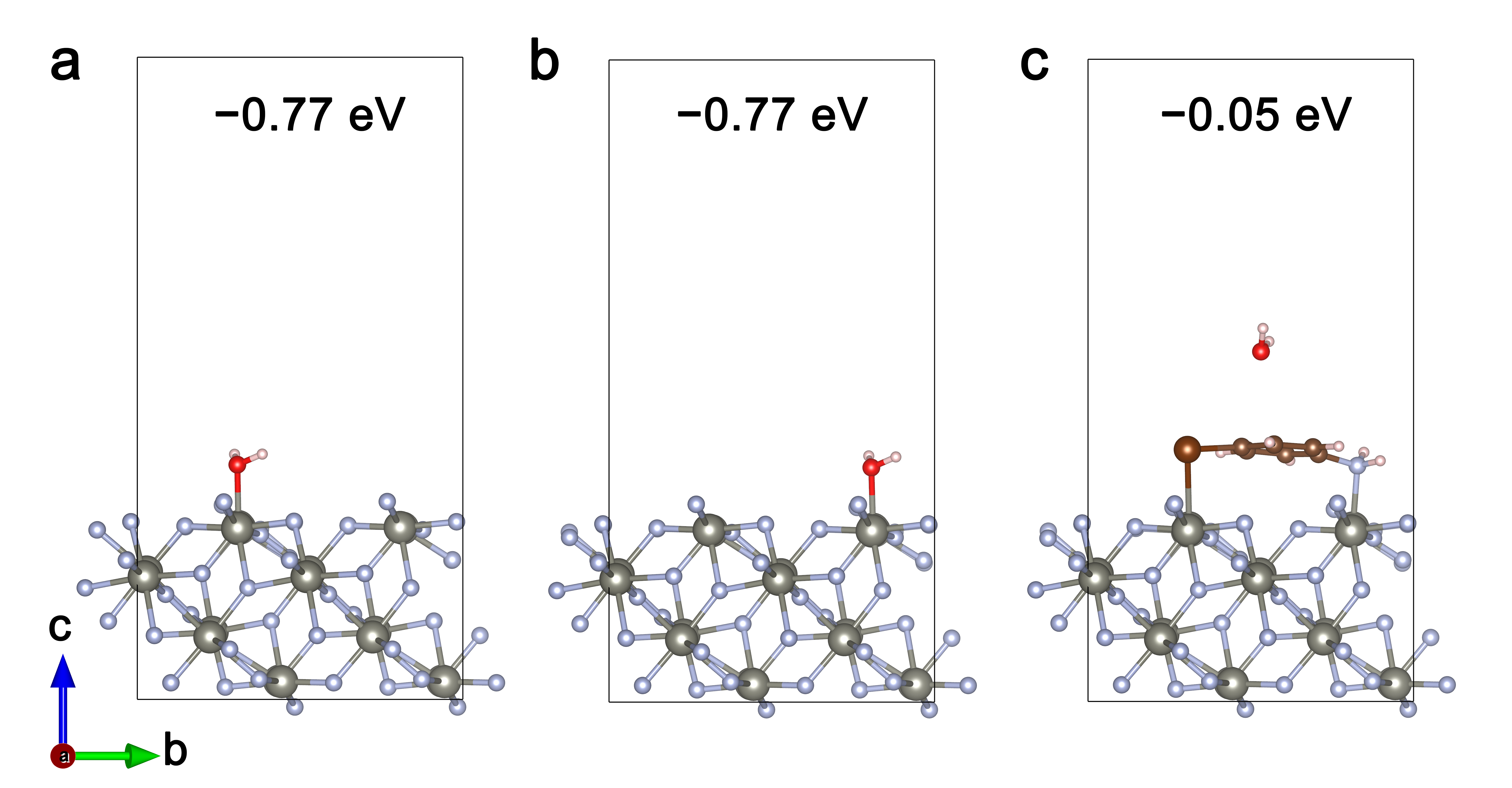


Figure S12. Adsorption configurations of H_2_O on pristine and PBA-covered ZnF_2_ (211) surfaces. (a,b) H_2_O adsorption on bare ZnF_2_ (211) with −0.77 eV. (c) Adsorption of H_2_O on the PBA-covered ZnF_2_ (211) surface with a dramatically weakened interaction (−0.05 eV).


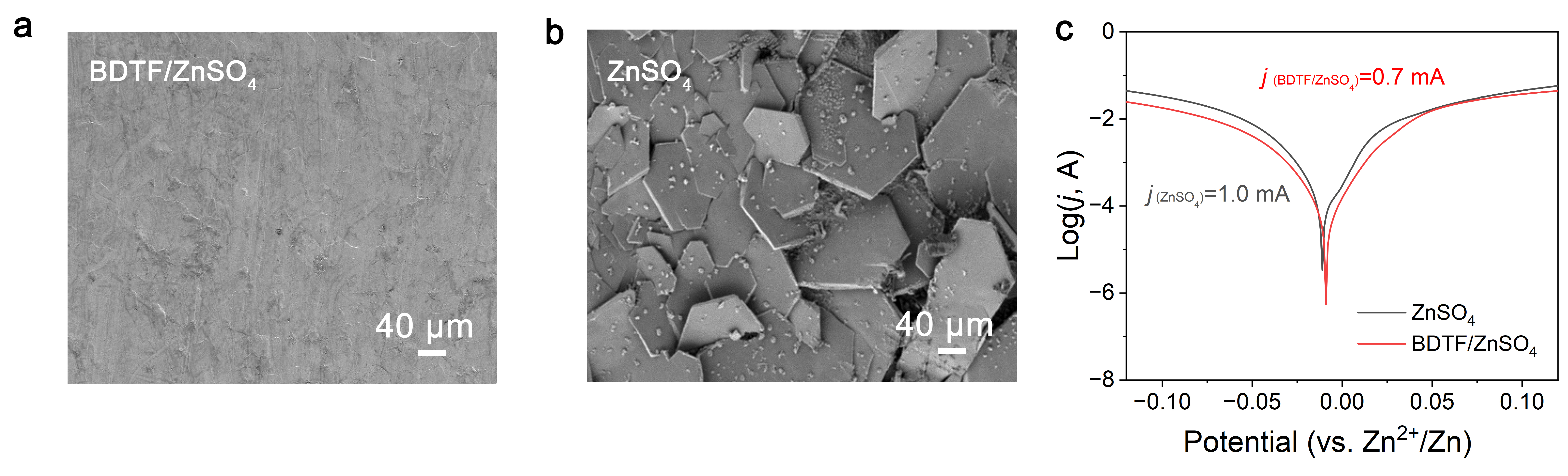


Figure S13. SEM images of cycled Zn after soaking in (a) BDTF/ZnSO_4_ and (b) ZnSO_4_ electrolytes. **(c) Tafel plots.**


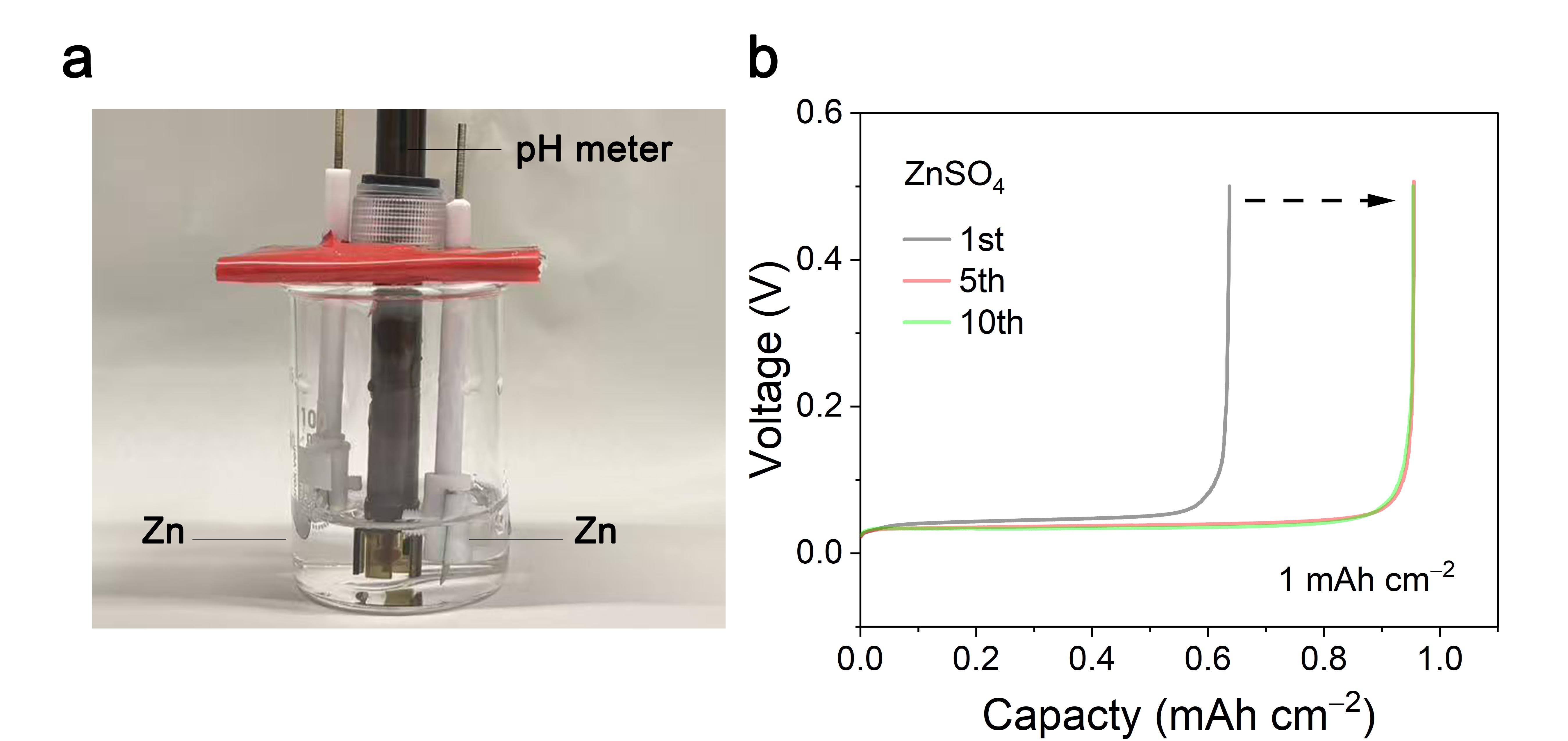


Figure S14. (a) *In situ* pH measurement setup for the Zn symmetric cells. (b) Voltage profiles of the 1st, 5th, and 10th cycles in ZnSO_4_ electrolyte.


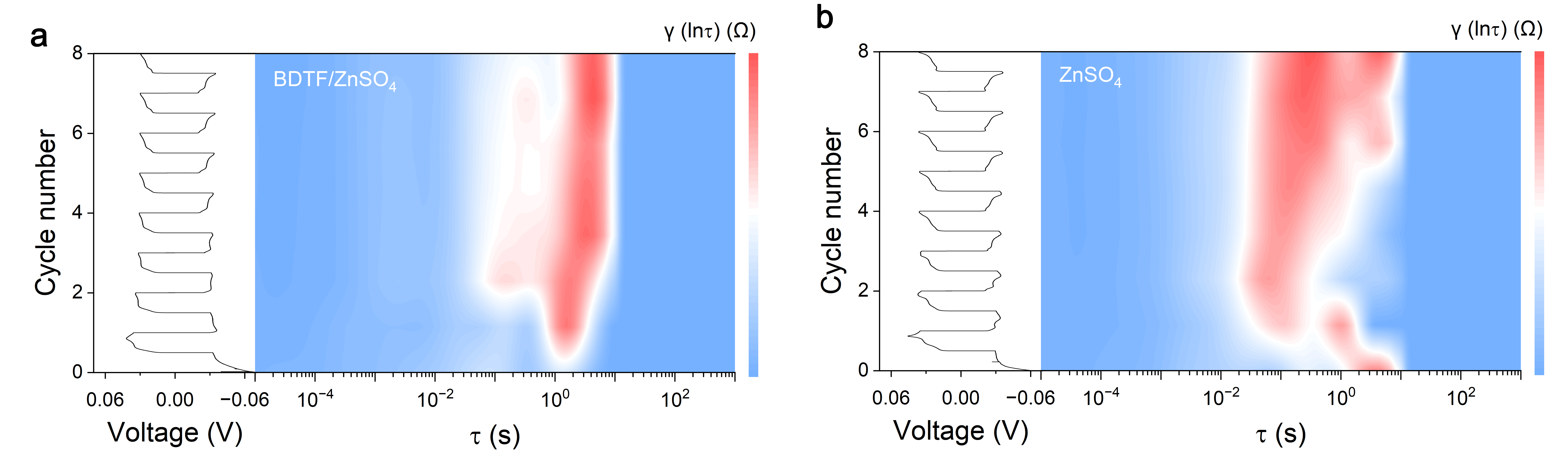


Figure S15. ***In situ* impedance evolution contour maps for Zn symmetric cells in (a) BDTF/**ZnSO_4_ **and (b)** ZnSO_4_ **electrolytes.**


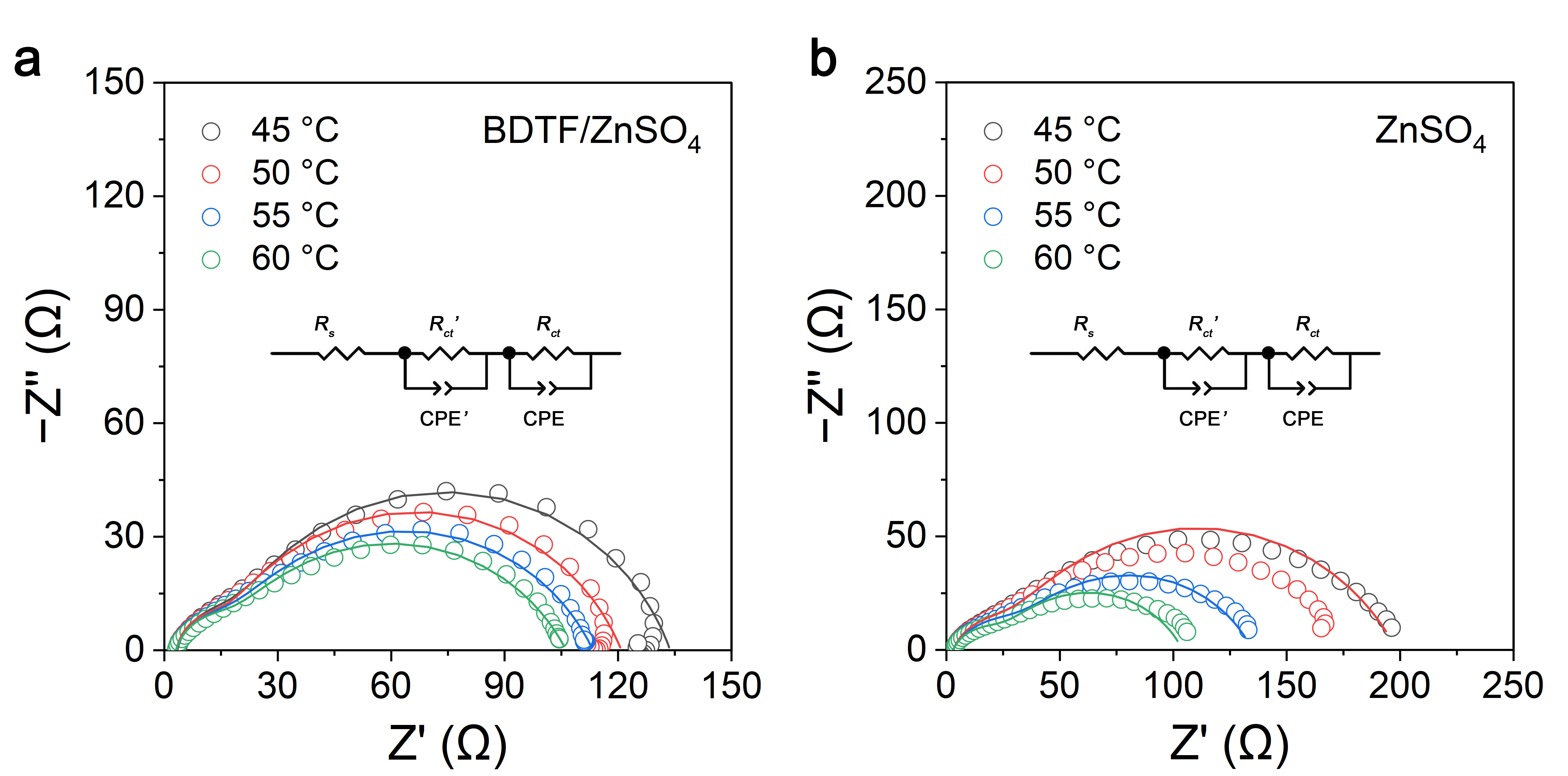


Figure S16. Nyquist plots of Zn symmetric cells with both electrolytes at different temperatures. **(a)** BDTF/ZnSO_4_. **(b)** ZnSO_4_.


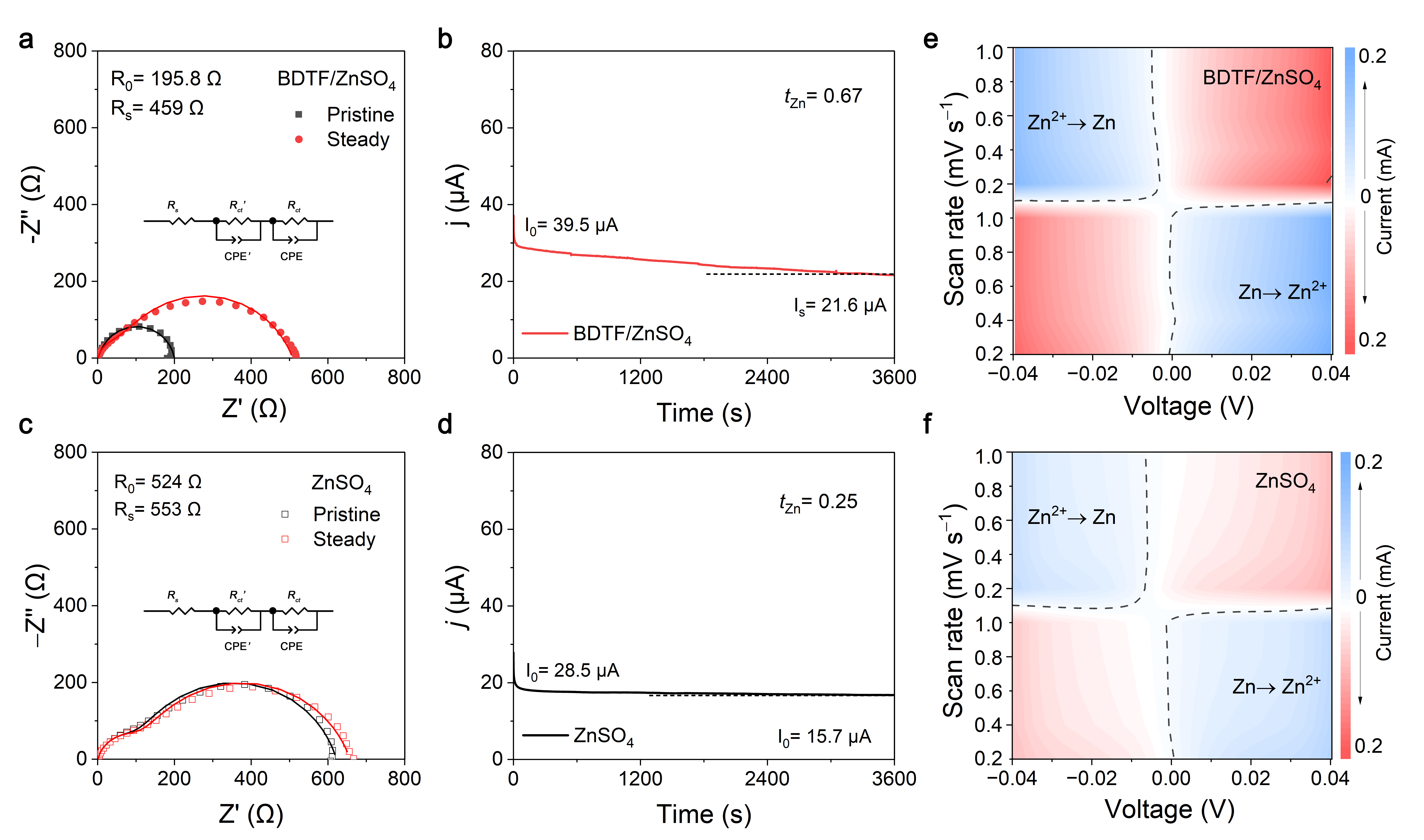


Figure S17. EIS curves before and after polarization with (a) BDTF/ZnSO_4_ and (c) blank ZnSO_4_ system. Chronoamperometry curves with a constant voltage polarization of 20 mV with (b) BDTF/ZnSO_4_ and (d) blank ZnSO_4_ system. CV curves of Zn plating/stripping with (e) BDTF-containing and (f) blank ZnSO_4_ electrolytes.


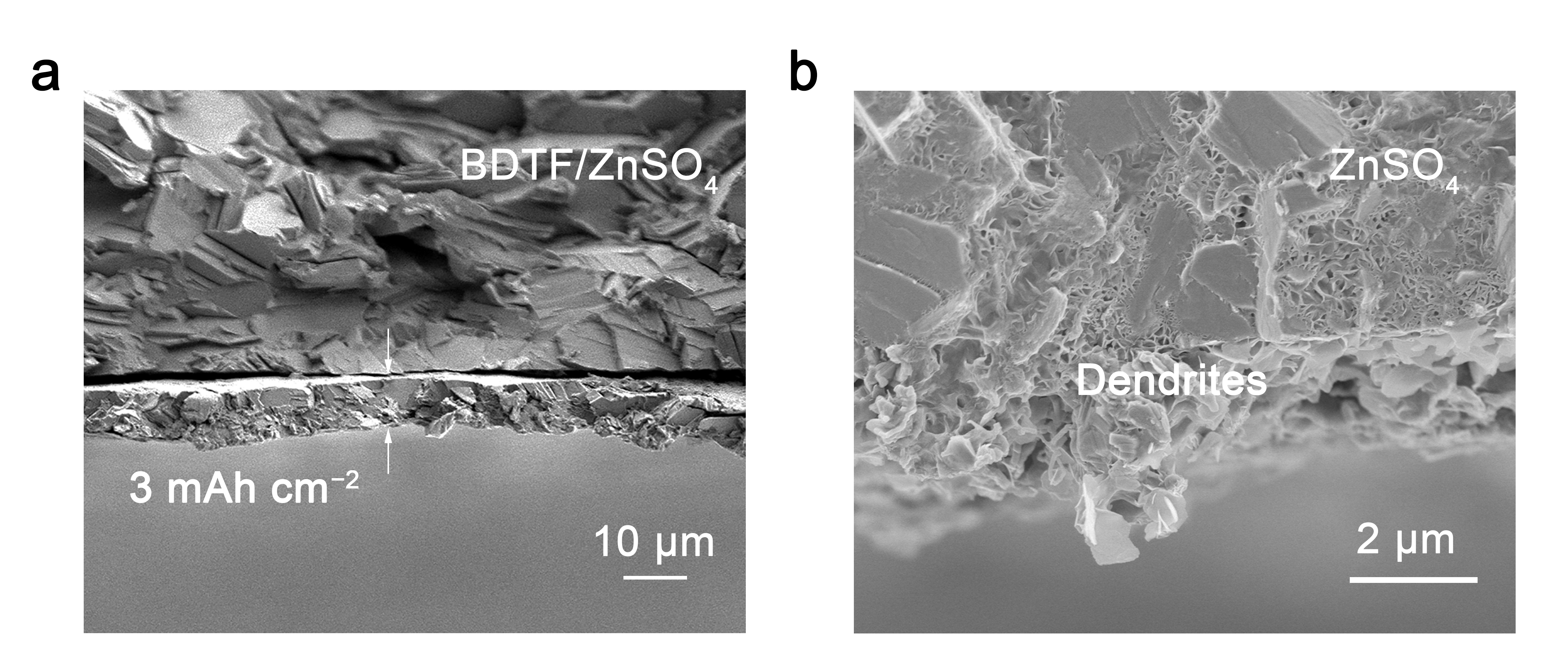


Figure S18. Cross-sectional SEM images of the Zn anode after deposition: **(a)** BDTF-containing electrolyte; **(b)** blank electrolyte.


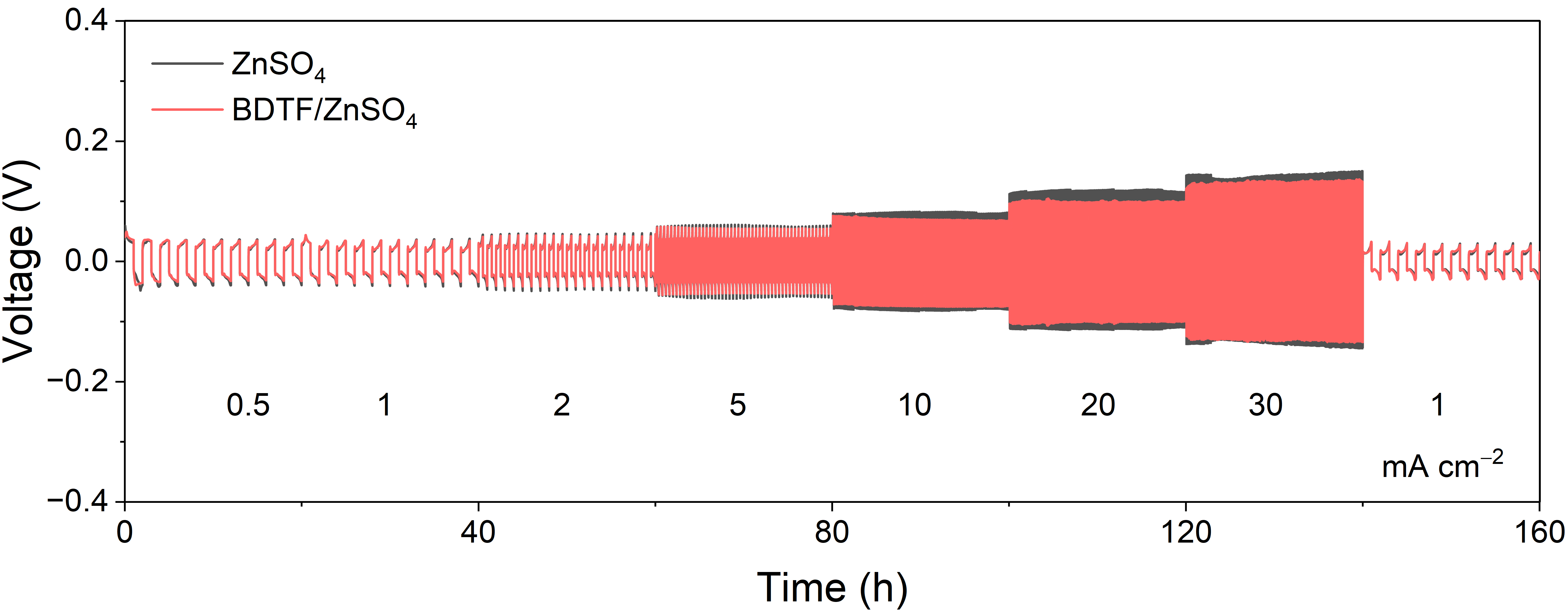


Figure S19. Voltage profiles of Zn symmetric cells under sequential current increase from 0.5 to 30 mA cm^−2^.


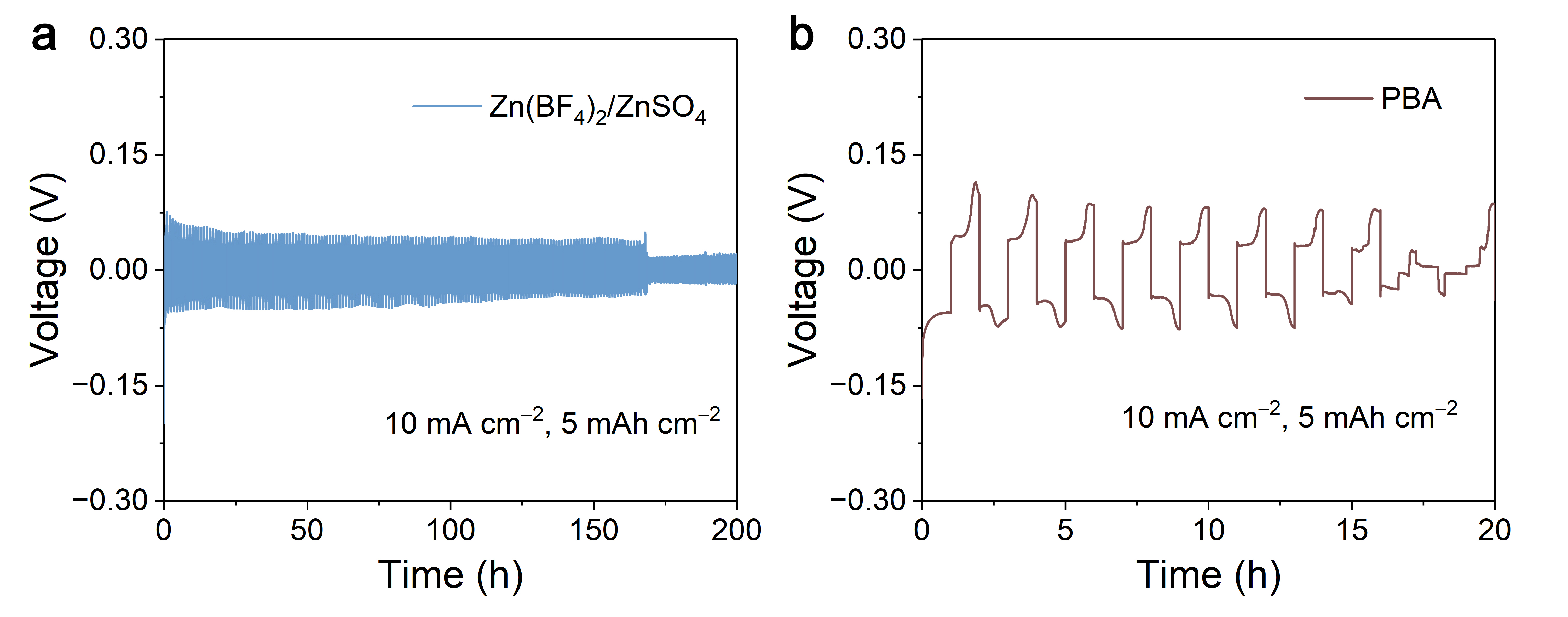


Figure S20. Cycling stability of the symmetric cell with **(a)** the Zn(BF_4_)_2_ additive and **(b)** the PBA layer-coated Zn electrodes.


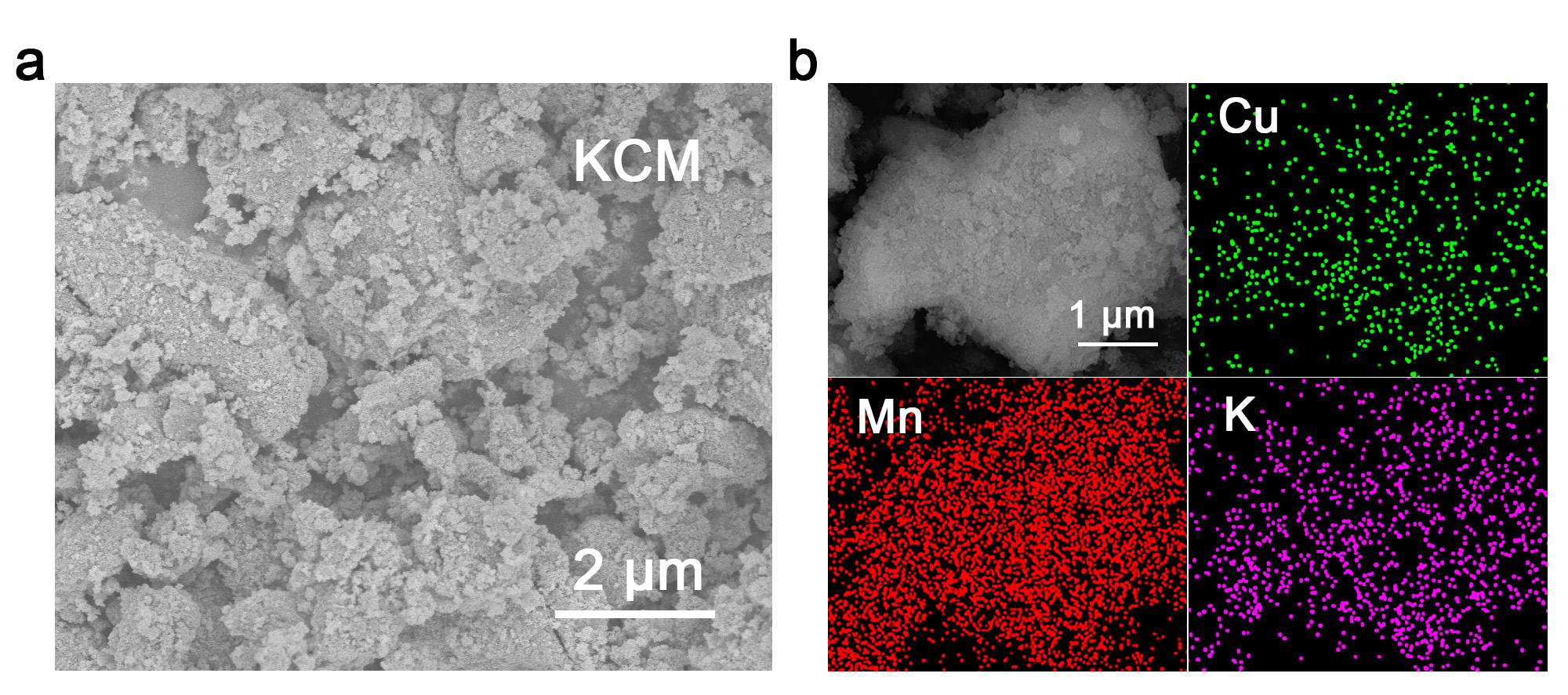


Figure S21. **(a)** SEM images and **(b)** EDS mapping of the KCM cathode.


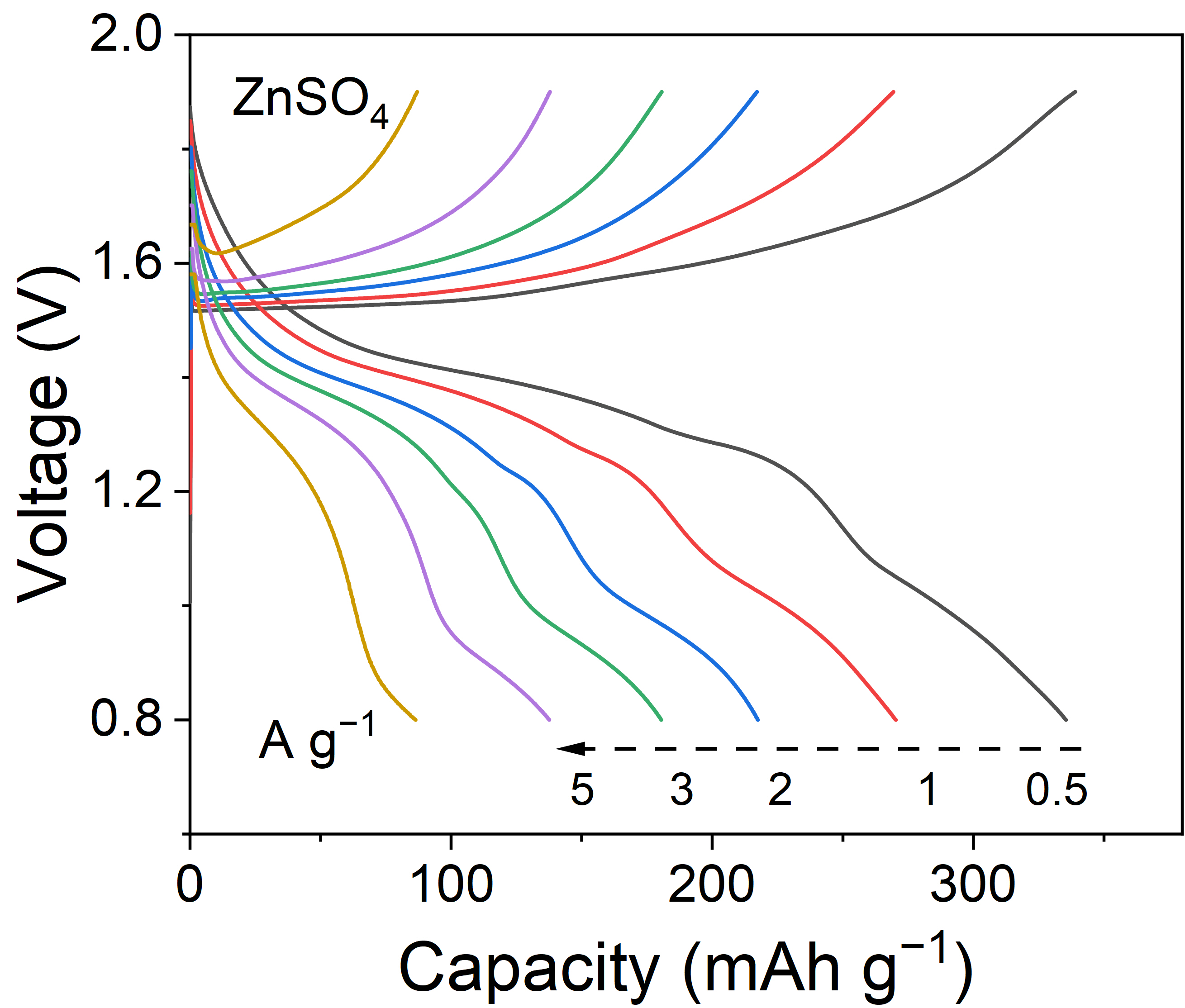


Figure S22. Voltage profiles across varying rates of the full cell without additives.


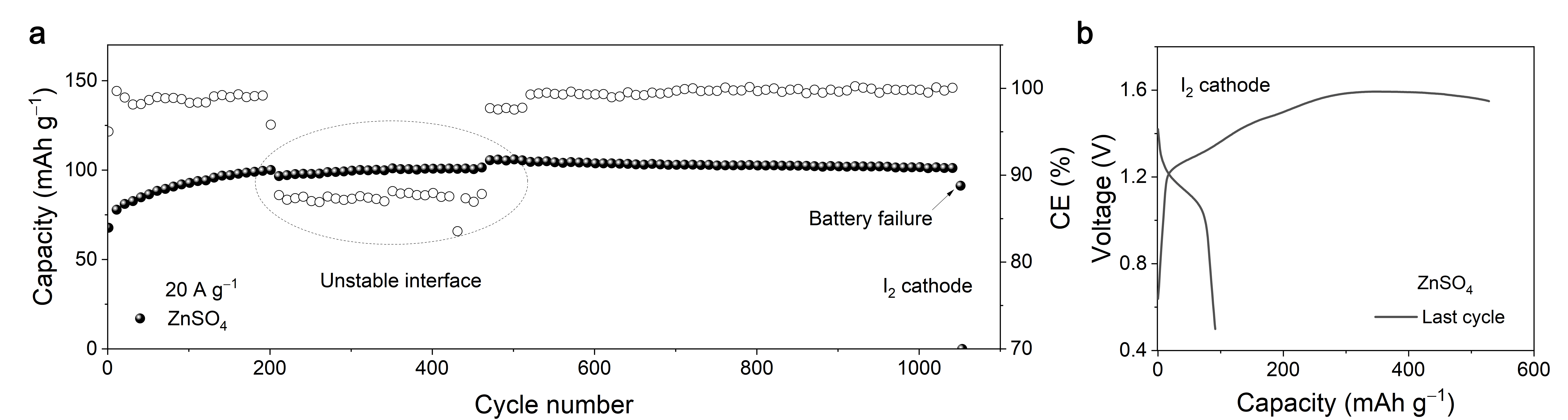


Figure S23. (a) Cycling stability of Zn|I_2_ cells without BDTF additive. (b) Corresponding charge–discharge profile at the last cycle without BDTF additive.


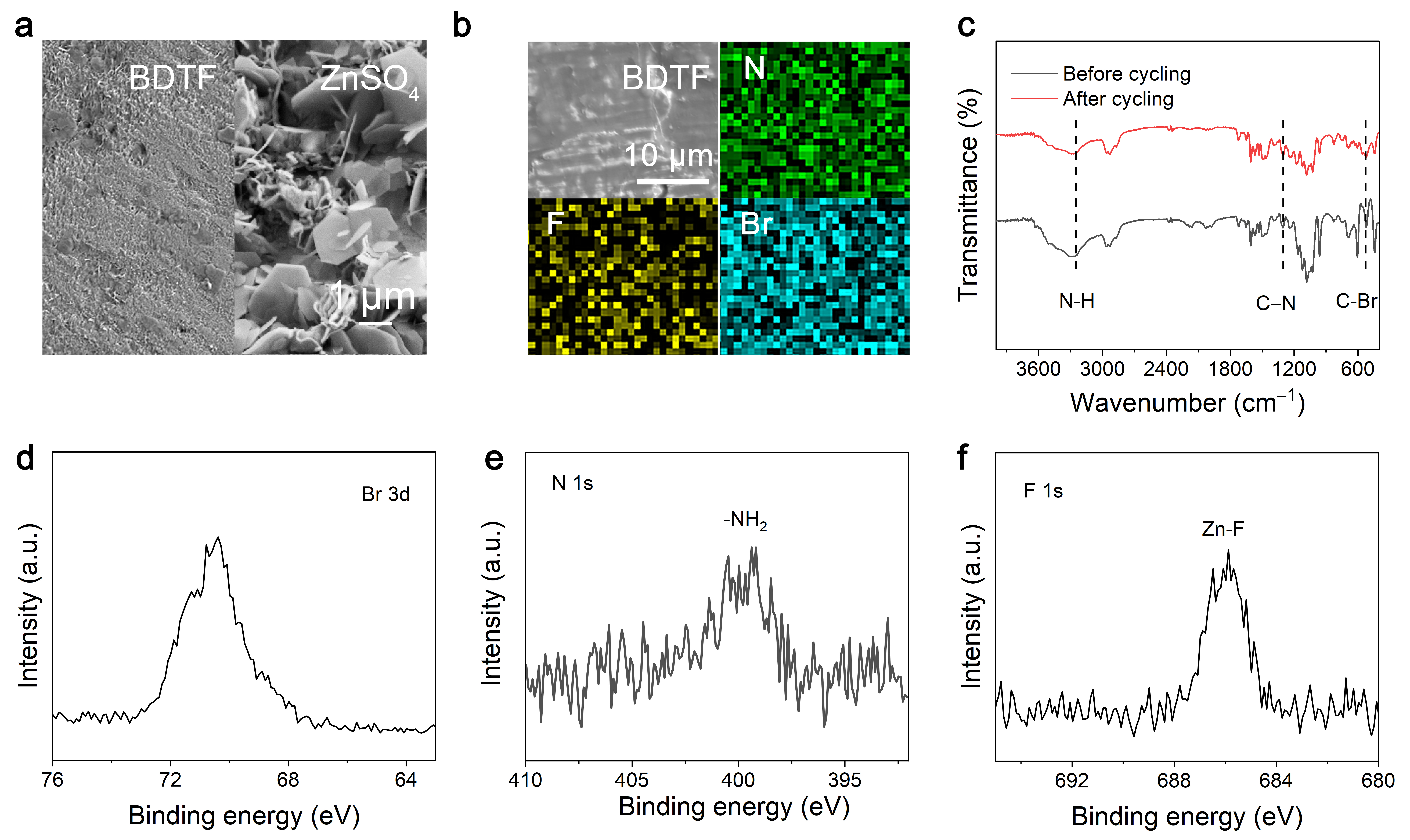


Figure S24. Surface characterization of the Zn anode after 500 cycles in the BDTF/ZnSO_4_ and blank ZnSO_4_ electrolyte. (a) SEM images. (b) EDS elemental mappings of N, F, and Br, showing the uniform distribution of the hybrid SEI components across the Zn surface. (c) ATR-FTIR spectra of the SEI before and after cycling. (d–f) High-resolution XPS spectra of (d) Br 3d, (e) N 1s, and (f) F 1s regions.


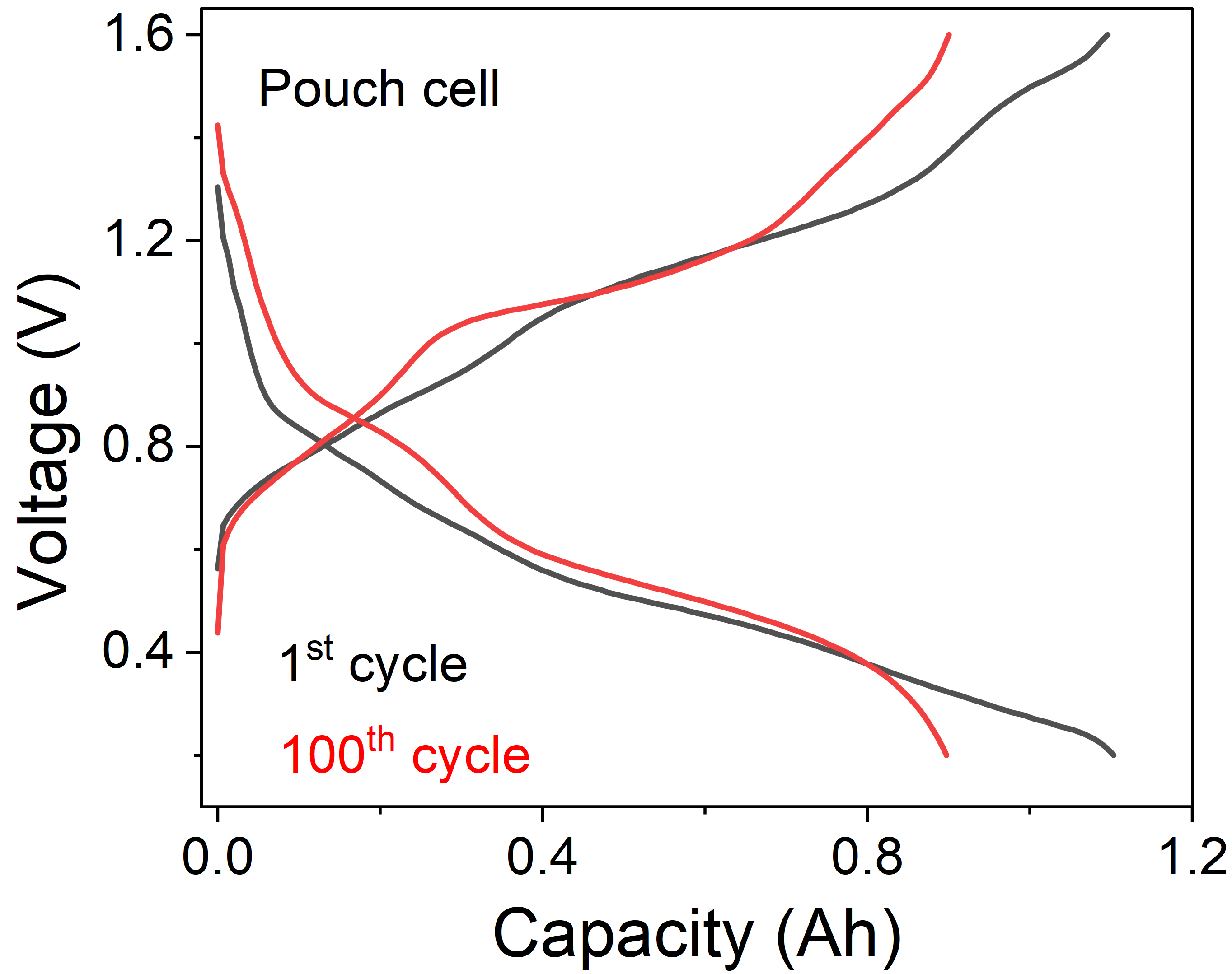


Figure S25. Voltage profiles of the Ah-level pouch cell with BDTF additive.


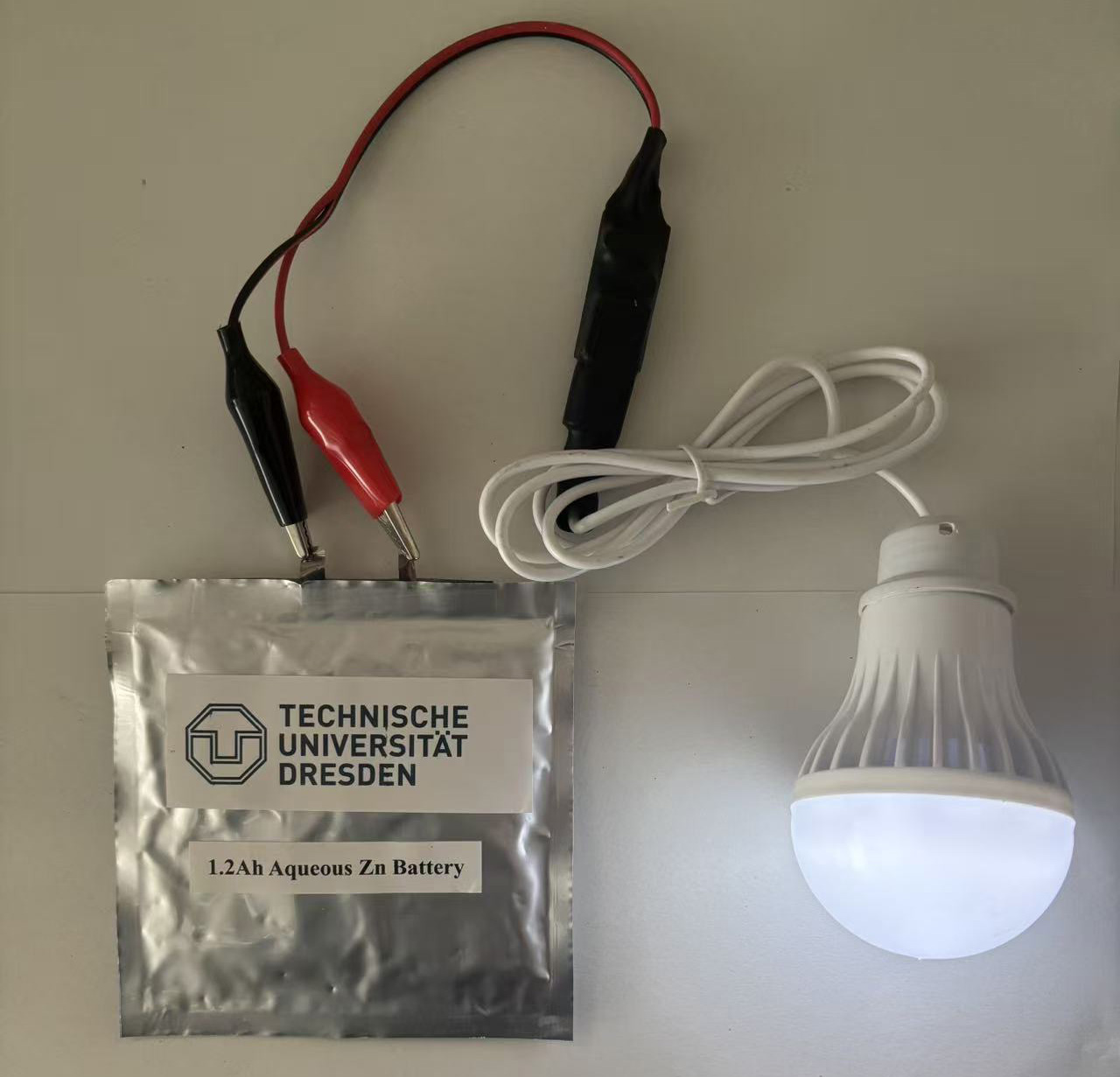


Figure S26. Photograph of a 1.2 Ah aqueous Zn pouch cell, demonstrating its practical capability by powering a commercial LED bulb.

**Table S1**. Comparison between conventional bilayer/hybrid SEIs and the electron-programmed molecular-lock SEI in this work.

| Aspect | Representative *in situ* hybrid SEIs | Molecular-lock SEI |
| --- | --- | --- |
| Method | Electrolyte/additive **decomposition–driven** SEI formation and composition tuning by solvation regulation or byproduct accumulation | Electron-triggered, **surface-confined molecular programming** at Zn interface |
| Structure | **Composition-driven** organic/inorganic coexistence without defined interfacial bonding | Bonding-defined interphase stabilized by molecular anchoring (**N–Zn coordination)** and electronic polarization **(σ-hole effect)** |
| Functional mechanism | Chemical passivation and suppression of parasitic reactions by **compositional tuning** | **Dipole-mediated electrostatic reorganization** that **stabilizes ZnF_2_** and guides Zn^2+^ transport |
| References | ^[6–13]^ | Our work |

**Table S2**. EXAFS fitting parameters at the Zn K-edge for SEI layers (*Ѕ*_0_^2^=0.83 from Zn-foil).

|  | shell | CN*^a^* | R*^b^*(Å) | σ^2^*^c^*(Å^2^) | ΔE_0_*^d^*(eV) | R factor |
| --- | --- | --- | --- | --- | --- | --- |
| SEI | Zn-F | 4.26±0.7 | 2.00±0.03 | 0.0090 | 3.7±0.8 | 0.013 |
|  | Zn-Br | 1.08±0.3 | 2.84±0.01 | 0.0091 |  |  |

*^a^CN*: coordination numbers; *^b^R*: bond distance; *^c^σ*^2^: Debye-Waller factors; *^d^* Δ*E*_0_: the inner potential correction. R factor: goodness of fit. Error bounds that characterize the structural parameters obtained by EXAFS spectroscopy were estimated as CN±20%, R ± 1%, and σ^2^ ± 20%.

**Table S3**. Fitting results of *R*_ct_ at different temperatures for the symmetric cell with the BDTF additive.

| Temperature | 35 ℃ | 40 ℃ | 45 ℃ | 50 ℃ |
| --- | --- | --- | --- | --- |
| *R*_ct_ (Ω) | 117.3 | 107 | 98 | 91 |
| Error (%) | 5.99 | 4.47 | 5 | 2.69 |

**Table S4**. Fitting results of *R*_ct_ at different temperatures for the symmetric cell without the BDTF additive.

| Temperature | 35 ℃ | 40 ℃ | 45 ℃ | 50 ℃ |
| --- | --- | --- | --- | --- |
| *R*_ct_ (Ω) | 171 | 144 | 105 | 80 |
| Error (%) | 4.46 | 6.59 | 6.97 | 1.95 |

**Table S5**. Fitting results of *R*_ct_ for the symmetric cells before and after the chronoamperometry test.

| Samples | | *R*_ct_ | Error (%) |
| --- | --- | --- | --- |
| BDTF/ZnSO_4_ | Before | 195.8 | 4.15 |
|  | After | 459 | 5.59 |
| ZnSO_4_ | Before | 524 | 1.02 |
|  | After | 553 | 8.92 |

**Table S6**. Comparison of representative electrolyte additives for aqueous Zn batteries reported in previous literature and this work. Key performance metrics include additive concentration, cycling stability, depth of discharge (DOD), current density (J), and areal capacity.

| Electrolyte Additives | Concentration (M) | Cycle number | DOD% | J (mA cm^−2^) | Capacity (mAh cm^−2^) | References |
| --- | --- | --- | --- | --- | --- | --- |
| BDTF | 0.001 | 101.4 | 80 | 10 | 7.4 | This work |
| D-Valine | 0.05 | 12.8 | 74 | 1 | 7 | ^[14]^ |
| D-Arabinose | 0.1 | 3.1 | 76.6 | 1 | 40 | ^[15]^ |
| Octyltrimethyl ammonium bromide | 0.5 | 64.1 | 20 | 1 | 2.34 | ^[16]^ |
| IDAN | 0.0526 | 45 | 90 | 1.06 | 5.3 | ^[17]^ |
| 1, 4-diol | 0.15 | 4.4 | 67.7 | 1 | 40 | ^[18]^ |
| PCA-Na | 0.823 | 23.9 | 93 | 5 | 27.2 | ^[19]^ |
| BmBr | 0.05 | 8 | 85 | 1 | 20 | ^[20]^ |
| BHED | 0.004 | 87.5 | 51 | 3 | 3 | ^[21]^ |
| PVP | 0.18 | 80 | 70 | 4.1 | 4.1 | ^[22]^ |
| TCEP | 0.001 | 10 | 85 | 1 | 20 | ^[23]^ |

**Table S7**. Comparison of representative full-cell configurations for aqueous Zn batteries reported in previous literature and this work. Key performance metrics include cathode material, stabilization strategy, current density (J), cycling life, and capacity retention.

| Cathode | Strategies | J (A g^−1^) | Cycles | Retention (%) | References |
| --- | --- | --- | --- | --- | --- |
| KCM | Additive | 10 | 2000 | 99.3 | This work |
| MnO_2_ | COF SEI | 2 | 2000 | 75.6 | ^[24]^ |
| MnO_2_ | glutaric acid additive | 2 | 1000 | 88.2 | ^[25]^ |
| NVO | O-phthalaldehyde SEI | 5 | 1000 | 71 | ^[26]^ |
| Zn_0.25_V_2_O_5_·nH_2_O | ZnS/ZnF_2_ SEI | 0.5 | 1200 | 91.57 | ^[27]^ |
| NH_4_V_4_O_10_ | Maltitol additive | 2 | 500 | 80 | ^[28]^ |
| MnO_2_ | Vermiculite SEI | 1 | 500 | 61 | ^[29]^ |
| MnO_2_ | Hybrid additive | 1 | 2000 | 75.6 | ^[30]^ |
| NVO | Metformin hydrochloride additive | 2 | 500 | 83.2 | ^[31]^ |
| VO_2_ | Hybrid ZnS/ZnCO_3_ SEI | 0.1 | 300 | 91.1 | ^[32]^ |
| V_2_O_5_ | Gel electrolyte | 1 | 1000 | 85.2 | ^[33]^ |
| MnO_2_ | DMSO additive | 0.5 | 395 | 70 | ^[34]^ |

**Table S8**. Comparison of aqueous Zn pouch cell performances using various cathodes and electrolytes.

| Cathode | Electrolyte | Cathode loading (mg cm^−2^) | Capacity (Ah) | Cycle number | Retention (%) | References |
| --- | --- | --- | --- | --- | --- | --- |
| **ZnVO** | **BDTF/ZnSO_4_** | **21** | **1.2** | **100** | **81** | **This work** |
| ZnVO | CarraChi/ZnSO_4_ | 15 | 0.9 | 200 | 84 | ^[35]^ |
| NH_4_V_4_O_10_ | ZnSO_4_ | 10 | 0.028 | 80 | 84.1 | ^[36]^ |
| KMO | ZnSO_4_ | 6.7 | 0.56 | 50 | 84.3 | ^[37]^ |
| VO_2_ | PD-ZnSO_4_ | 8 | 0.13 | 50 | 61 | ^[32]^ |
| VOH | 1M Zn(TFSI)_2_/ sulfolane | 4 | 0.2 | 55 | 81.6 | ^[38]^ |
| VOPO_4_ | 4M Zn(OTf)_2_ + 0.5M Me_3_EtNOTf | 3 | 0.05 | 1000 | 80 | ^[39]^ |
| ZnC_2_O_4_·2H_2_O | CH_3_COOLi | 23 | 1.1 | 40 | 88 | ^[40]^ |
| NVO | Zn(OTf)_2_/TG4/H_2_O | 9.02 | 1.3 | 70 | 60 | ^[41]^ |
| MnO_2_ | ZnSO_4_-FS | 15 | 0.5 | 100 | 81 | ^[42]^ |

**Table S9**. Summary of component parameters and calculated gravimetric and volumetric energy densities of the 1.232 Ah Zn|ZnVO pouch cell.

| **Component** | **Value** | **Notes** |
| --- | --- | --- |
| Effective Electrode Area | 64 cm^2^ (8×8 cm) ×5 | Projected electrode area |
| Cathode Active Material | 6.72 g | ZnVO |
| Carbon Additive | 1.92 g | SuperP |
| Binder | 0.96 g | PVDF |
| Zn Foil | 6.735 g | 100 µm × 3 pieces |
| Carbon Cloth | 3.90 g | 5 sheets |
| Electrolyte | 3.307 g | 2.5 mL |
| Separator | 1.175 g | Bacterial cellulose membrane |
| Al–Plastic Pouch | 4.638 g | Mass |
| Tabs | 0.450 g | 2 Ni tabs |
| Ti foil | 0.160 g | Connector |
| Total Mass | 29.965 g (0.029965 kg) | Summed mass of all parts |
| Discharge Capacity | 1.232 Ah | Maximum measured capacity |
| Voltage Plateau | 0.9 V | Used for energy calculation |
| Discharge Energy | 1.108 Wh | Capacity × Voltage |
| Electrolyte/Capacity (E/C) | 2.69 g·Ah^−1^ | Electrolyte mass per Ah |
| Cell Volume | 37.44 cm^3^ (0.03744 L) | Electrode stack only |

$$\mathrm{Gravimetric}Energy density= \frac{1.108 Wh}{29.965 g}=37 Wh {kg}^{-1}(based on pouch full cell)$$

$$\mathrm{Gravimetric}Energy density= \frac{1.108 Wh}{25.327 g}=43.7 Wh {kg}^{-1} (based on full cell except pouch)$$

$$\mathrm{Gravimetric}Energy density= \frac{1.108 Wh}{6.72 g}=165 Wh {kg}^{-1} (based on ZnVO)$$

$$\mathrm{Volumetric}Energy density= \frac{1.108 Wh}{0.03744 L}=29.6 Wh L^{-1}$$

**References**

[1] X. Gao, C. Shen, H. Dong, Y. Dai, P. Jiang, I. P. Parkin, H. Zhang, C. J. Carmalt, G. He, Co-Intercalation Strategy for Simultaneously Boosting Two-Electron Conversion and Bulk Stabilization of Mn-Based Cathodes in Aqueous Zinc-Ion Batteries. *Energy Environ. Sci.* **2024**, *17*, 2287.

[2] G. Kresse, J. Furthmüller, Efficient Iterative Schemes for *Ab Initio* Total-Energy Calculations Using a Plane-Wave Basis Set. *Phys. Rev. B* **1996**, *54*, 11169.

[3] P. Hohenberg, W. Kohn, Inhomogeneous Electron Gas. *Phys. Rev.* **1964**, *136*, B864.

[4] J. P. Perdew, K. Burke, M. Ernzerhof, Generalized Gradient Approximation Made Simple. *Phys. Rev. Lett.* **1996**, *77*, 3865.

[5] J. Shi, G. Wang, D. Tian, X. Hai, R. Meng, Y. Xu, Y. Teng, L. Ma, S. Xi, Y. Yang, X. Zhou, X. Fu, H. Li, Q. Cai, P. He, H. Lin, J. Chen, J. Li, J. Li, Q. He, Q.-H. Yang, J. Li, D. Wu, Y.-G. Wang, J. Wu, J. Lu, Defying the Oxidative-Addition Prerequisite in Cross-Coupling through Artful Single-Atom Catalysts. *Nat. Commun.* **2025**, *16*, 3223.

[6] D. Ma, F. Li, K. Ouyang, Q. Chen, J. Zhao, M. Chen, M. Yang, Y. Wang, J. Chen, H. Mi, C. He, P. Zhang, An Electrochemically Driven Hybrid Interphase Enabling Stable Versatile Zinc Metal Electrodes for Aqueous Zinc Batteries. *Nat. Commun.* **2025**, *16*, 4817.

[7] Z. Zhang, X. Lan, G. Liao, W. Du, Y. Zhang, M. Ye, Z. Wen, Y. Tang, X. Liu, C. C. Li, Coupling Zn^2+^ Ferrying Effect with Anion–π Interaction to Mitigate Space Charge Layer Enables Ultra‐high Utilization Rate Zn Anode. *Angew. Chem., Int. Ed.* **2025**, *64*, e202503396.

[8] Q. Zong, R. Li, J. Wang, Q. Zhang, A. Pan, Tailoring the Whole Deposition Process from Hydrated Zn^2+^ to Zn^0^ for Stable and Reversible Zn Anode. *Angew. Chem., Int. Ed.* **2024**, *63*, e202409957.

[9] Y. Zhao, Z. Chen, X. Gao, H. Dong, X. Zhao, G. He, H. Yang, In‐situ Self‐respiratory Solid‐to‐hydrogel Electrolyte Interface Evoked Well‐distributed Deposition on Zinc Anode for Highly Reversible Zinc‐ion Batteries. *Angew. Chem., Int. Ed.* **2024**, *64*, e202415251.

[10] G. Zeng, Q. Sun, S. Horta, P. R. Martínez-Alanis, P. Wu, J. Li, S. Wang, M. Ibáñez, Y. Tian, L. Ci, A. Cabot, Modulating the Solvation Structure to Enhance Amorphous Solid Electrolyte Interface Formation for Ultra-Stable Aqueous Zinc Anode. *Energy Environ. Sci.* **2025**, *18*, 1683.

[11] X. Yang, X. Tang, J. Lei, X. Zeng, J. Wen, A. Liu, S. Xia, Q. Luo, J. Liu, A. Xue, D. Han, G. Zhou, A Fluorine‐free Organic/Inorganic Interphase for Highly Reversible Aqueous Zinc Batteries. *Angew. Chem., Int. Ed.* **2025**, e202504003.

[12] G. Yin, H. Wang, M. Zhou, T. Long, M. Ding, B. Xie, X. Wu, J. Li, W. Ling, J. Dai, X. Zeng, Inhibiting Interfacial Electron Leakage via an Artificial Rectified Layer for Longevous Zinc Metal Anodes. *Angew. Chem., Int. Ed.* **2025**, *64*, e202423244.

[13] W. Yang, S. Zhang, J. Gao, R. Zhu, H. Li, Q. Zhang, D. Wang, H. Yang, F. Gao, H. Zhou, Nucleophilic Substitution Enables Robust Fluorinated Interphase for Low *N* /*P* Ratio Zinc Battery. *Angew. Chem., Int. Ed.* **2026**, e21414.

[14] J. Lin, C. Ji, G. Guo, Y. Luo, P. Huang, L. Sun, F. Xu, W. Pfleging, K. S. Novoselov, Interfacial H‐bond Network/Concentration Fields/Electric Fields Regulation Achieved by D‐valine Anions Realizes the Highly Efficient Aqueous Zinc Ion Batteries. *Angew. Chem., Int. Ed.* **2025**, *64*, e202501721.

[15] Y. Yang, Y. Li, Q. Zhu, B. Xu, Optimal Molecular Configuration of Electrolyte Additives Enabling Stabilization of Zinc Anodes. *Adv. Funct. Mater.* **2024**, *34*, 2316371.

[16] D. Tang, X. Zhang, D. Han, C. Cui, Z. Han, L. Wang, Z. Li, B. Zhang, Y. Liu, Z. Weng, Q. Yang, Switching Hydrophobic Interface with Ionic Valves for Reversible Zinc Batteries. *Adv. Mater.* **2024**, *36*, 2406071.

[17] R. Zhang, T. Shui, A. Li, H. Xia, G. Xu, L. Ji, C. Lu, W. Zhang, Z. Sun, Novel *in Situ* SEI Fabrication on Zn Anodes for Ultra-High Current Density Tolerance Enabled by Electrical Excitation–Conjugation of Iminoacetonitriles. *Energy Environ. Sci.* **2025**, *18*, 1011.

[18] X. Ren, G. Chen, P. Chang, S. Ju, Y. Wu, Crystal Plane Shielding and D-Band Modulation Synergistically Achieve Durable (100) Textured Zinc Anodes. *Energy Environ. Sci.* **2025**, *18*, 1867.

[19] K. Ouyang, S. Chen, L. Yu, H. Qin, A. Liu, Y. Liu, Q. Wu, B. Ran, S. Wei, F. Gao, K. Zhang, J. Hu, Y. Huang, An Electrochemically Paralleled Biomass Electrolyte Additive Facilitates the Integrated Modification of Multi-Dimensional Zn Metal Batteries. *Energy Environ. Sci.* **2025**, *18*, 4416.

[20] Y. Lv, C. Huang, M. Zhao, M. Fang, Q. Dong, W. Tang, J. Yang, X. Zhu, X. Qiao, H. Zheng, C. Sun, L. Zheng, M. Zheng, Y. Xu, J. Lu, Synergistic Anion–Cation Chemistry Enables Highly Stable Zn Metal Anodes. *J. Am. Chem. Soc.* **2025**, *147*, 8523.

[21] S. Han, M. Li, Q. Fan, Z. Han, X. Ming, W. Wang, W. Cai, H. Niu, Multifunctional Additives with Dynamic Sacrificial S–S Bonds for Building Self-Assembled Monolayers of Zn-Ion Batteries with Improved Stability and Longevity. *Energy Environ. Sci.* **2025**, *18*, 4186.

[22] R. Chen, Y. Zhong, P. Jiang, H. Tang, F. Guo, Y. Dai, J. Chen, J. Wang, J. Liu, S. Wei, W. Zhang, W. Zong, F. Zhao, J. Zhang, Z. Guo, X. Wang, G. He, Untangling the Role of Capping Agents in Manipulating Electrochemical Behaviors toward Practical Aqueous Zinc‐ion Batteries. *Adv. Mater.* **2025**, 2412790.

[23] M. Zhao, Y. Lv, Y. Xu, H. Yang, Z. Bo, J. Lu, Ordered Zinc Electrodeposition from Single-Crystal Units to Polycrystalline Stacking within Solid-Electrolyte Interphase in Battery Anodes. *Nat. Commun.* **2025**, *16*, 2843.

[24] S. Zhang, J. Chen, W. Chen, Y. Su, Q. Gou, R. Yuan, Z. Wang, K. Wang, W. Zhang, X. Hu, Z. Zhang, P. Wang, F. Wan, J. Liu, B. Li, Y. Wang, G. Zheng, M. Li, J. Sun, Regulating Water Molecules via Bioinspired Covalent Organic Framework Membranes for Zn Metal Anodes. *Angew. Chem., Int. Ed.* **2025**, *64*, e202424184.

[25] Y. Ouyang, W. Zong, X. Gao, S. X. Leong, J. R. T. Chen, Y. Dai, H. Dong, I. Y. Phang, P. R. Shearing, G. He, Y. Miao, T. Liu, X. Y. Ling, Regulating Interfacial Molecular Configuration to Drive Facet‐selective Zn Metal Deposition. *Angew. Chem., Int. Ed.* **2025**, *64*, e202504965.

[26] S. Wei, H. Shou, Z.-H. Qi, S. Chen, Y. Han, S. Shi, Y. Wang, P. Zhang, J. Shi, Z. Zhang, Y. Cao, C. Wang, J. Cui, X. Wu, Z. Liu, L. Song, In Situ Detection of the Molecule-Crowded Aqueous Electrode–Electrolyte Interface. *J. Am. Chem. Soc.* **2025**, *147*, 10943.

[27] Z. Wu, S. Yang, Z. Wei, Y. Wang, X. Yang, J. Zhu, H. Hong, P. Li, X.-F. Yu, C. Peng, C. Zhi, Constructing Lipid-like Biomimetic Structure via Electrolyte Designation for Stable Zinc-Ion Batteries. *ACS Nano* **2025**, *19*, 14085.

[28] Z. Xiao, X. Dai, J. Zhu, D. Liu, L. Liu, X. Liu, Y. Li, Z. Qian, R. Wang, Hydrogen Bond Competition Optimizing Aqueous Zn Ion Solvation and (002) Interfacial Deposition with Ultralong Stability. *Adv. Funct. Mater.* **2025**, 2424860.

[29] Z. Guo, Z. Liu, Y. Zhang, H. Li, M. Qi, C. Zhao, X. Zhang, Z. Wu, J. Yuan, N. Zhang, Ultrastrong Bioinspired “Brick-and-Mortar” Artificial SEI for Dendrite-Free Zn Anode. *Matter* **2025**, *8*, 102269.

[30] T. Xue, Y. Mu, Z. Zhang, J. Guan, J. Qiu, C. Yang, L. Zang, L. Zeng, Enhanced Zinc Deposition and Dendrite Suppression in Aqueous Zinc‐ion Batteries via Citric Acid‐aspartame Electrolyte Additives. *Adv. Energy Mater.* **2025**, *15*, 2500674.

[31] S. Zheng, Y. Wang, B. Luo, K. Zhang, L. Sun, Z. Bao, G. Duan, D. Chen, H. Hu, J. Huang, Z. Ye, Compacting Surface Charge Layer for Efficient Charge Transfer toward Stable Zn Anode. *Energy Environ. Sci.* **2025**, *18*, 5319.

[32] W. Zhang, S. Zhu, T. Yang, L. Wu, J. Li, J. Liang, Y. Liu, L. Cui, C. Tang, X. Chen, H. Zhou, F. Qiao, M. Zhou, P. Luo, F. Chi, X. Liao, L. Zhang, Q. An, Hydrogen/Electron Amphiphilic Bi‐functional Water Molecular Inactivator‐assisted Interface Stabilization in Highly Reversible Zn Metal Batteries. *Angew. Chem., Int. Ed.* **2025**, *64*, e202419732.

[33] J. Zhai, W. Zhao, L. Wang, J. Shuai, R. Chen, W. Ge, Y. Zong, G. He, X. Wang, Ultrathin Cellulosic Gel Electrolytes with a Gradient Hydropenic Interface for Stable, High-Energy and Flexible Zinc Batteries. *Energy Environ. Sci.* **2025**, *18*, 4241.

[34] X. Yu, M. Chen, J. Wang, S. Li, H. Zhang, Q. Zhao, H. Luo, Y. Deng, H. Liang, J. Zhou, F. Wang, D. Chao, Y. Zou, G. Feng, Y. Qiao, S.-G. Sun, Deciphering Multi-Dimensional Interfacial Mechanisms via Organic Cosolvent Engineering for Sustainable Zinc Metal Batteries. *Nat. Commun.* **2025**, *16*, 3820.

[35] F. Wang, J. Zhang, H. Lu, H. Zhu, Z. Chen, L. Wang, J. Yu, C. You, W. Li, J. Song, Z. Weng, C. Yang, Q.-H. Yang, Production of Gas-Releasing Electrolyte-Replenishing Ah-Scale Zinc Metal Pouch Cells with Aqueous Gel Electrolyte. *Nat. Commun.* **2023**, *14*, 4211.

[36] Y. Chen, S. Zhou, J. Li, X. Zhang, C. Zhou, X. Shi, C. Zhang, G. Fang, S. Liang, Z. Su, A. Pan, Tuning Zn^2+^ Deposition Kinetics towards Deep‐reversible Zinc Metal Batteries with All‐climate Adaptability. *Angew. Chem., Int. Ed.* **2025**, *64*, e202423252.

[37] R. Guo, X. Liu, F. Xia, Y. Jiang, H. Zhang, M. Huang, C. Niu, J. Wu, Y. Zhao, X. Wang, C. Han, L. Mai, Large‐scale Integration of a Zinc Metasilicate Interface Layer Guiding Well‐regulated Zn Deposition. *Adv. Mater.* **2022**, *34*, 2202188.

[38] M. Li, X. Wang, J. Hu, J. Zhu, C. Niu, H. Zhang, C. Li, B. Wu, C. Han, L. Mai, Comprehensive H_2_O Molecules Regulation via Deep Eutectic Solvents for Ultra-Stable Zinc Metal Anode. *Angew. Chem., Int. Ed.* **2023**, *62*, 202215552.

[39] L. Cao, D. Li, T. Pollard, T. Deng, B. Zhang, C. Yang, L. Chen, J. Vatamanu, E. Hu, M. J. Hourwitz, L. Ma, M. Ding, Q. Li, S. Hou, K. Gaskell, J. T. Fourkas, X.-Q. Yang, K. Xu, O. Borodin, C. Wang, Fluorinated Interphase Enables Reversible Aqueous Zinc Battery Chemistries. *Nat. Nanotechnol.* **2021**, *16*, 902.

[40] S. Guo, L. Qin, J. Wu, Z. Liu, Y. Huang, Y. Xie, G. Fang, S. Liang, Conversion-Type Anode Chemistry with Interfacial Compatibility toward Ah-Level near-Neutral High-Voltage Zinc Ion Batteries. *Natl. Sci. Rev.* **2024**, *11*, nwae181.

[41] S. Li, Y. Zhong, J. Huang, G. Lai, L. Li, L. Jiang, X. Xu, B. Lu, Y. Liu, J. Zhou, Regulating Interfacial Kinetics Boosts the Durable Ah-Level Zinc-Ion Batteries. *Energy Environ. Sci.* **2025**, *18*, 2599.

[42] R. Wang, J. Zhu, M. Yang, Z. Niu, Simultaneous Manipulation of Anions and Water Molecules by Lewis Acid–Base for Highly Stable Zn Anodes. *Angew. Chem., Int. Ed.* **2025**, *64*, e202501327.
